# Zirconium (IV) layered phosphonate-phosphate as catalysts for the valorization of glycerol

**DOI:** 10.3389/fchem.2025.1735925

**Published:** 2026-01-12

**Authors:** Nahal Ghanemnia, Martina Saitta, Elien Derveaux, Richa Tomer, Nick Gys, Tom Hauffman, Peter Adriaensens, Sophie Hermans, Carmela Aprile, Wouter Marchal

**Affiliations:** 1 UHasselt, Institute for Materials Research (IMO-IMOMEC), Analytical and circular Chemistry (ACC), Agoralaan, Diepenbeek, Belgium; 2 Laboratory of Applied Materials Chemistry, Unit of Nanomaterials Chemistry (UCNANO), Namur Institute of Structured Matter (NISM), Department of Chemistry, University of Namur, Namur, Belgium; 3 Institute of Condensed Matter and Nanosciences (IMCN), Université catholique de Louvain (UCLouvain), Louvain-la-Neuve, Belgium; 4 UHasselt, Institute for Materials Research (IMO-IMOMEC), Analytical and circular Chemistry (ACC), NMR group, Agoralaan, Diepenbeek, Belgium; 5 Centre for Membrane Separations, Adsorption, Catalysis, and Spectroscopy (cMACS), KU Leuven, Leuven, Belgium; 6 Sustainable Materials Engineering (SUME), Research Group of Electrochemical and Surface Engineering (SURF), Vrije Universiteit Brussel, Brussels, Belgium

**Keywords:** biomass valorization, glycerol ketalization, hybrid acid catalysts, metal phosphonate-phosphates, hybrid porous materials

## Abstract

The valorization of glycerol, a large-volume byproduct of biodiesel production, remains a key challenge for the biorefinery sector. Among the available strategies, its acetalization with acetone offers a sustainable route to produce solketal, a valuable fuel additive. The feasibility of this valorization route is, however, largely dependent on the continuous research towards highly performant, stable catalysts with carefully designed acidic sites. In this work, hybrid porous zirconium phosphonate-phosphate materials were synthesized and evaluated as recyclable heterogeneous catalysts. The acidity was systematically investigated using Hammett indicators, supported by solid-state ^31^P NMR, XPS, and ammonia TPD analyses. Their structural and thermal stability was also assessed. The incorporation of phosphate groups was found to be essential to provide sufficient Brønsted acidity and enhance the long-term stability of the catalysts, as evidenced by their successful recyclability during multiple catalytic runs. In addition, yields of 85%, with a selectivity of 98% can be reported in optimal conditions, and the catalyst was even found to offer very promising conversions at room temperature.

## 1 Introduction

Transportation is a key contributor to greenhouse gas emissions, spurring the development of biofuels and bio-based fuel additives in the last decades (Hoogendoorn and van Kasteren, 2010; Smith et al., 2015). Biodiesel production consists of the transesterification of triglycerides present in vegetable oil or animal fat (Wang et al., 2024). The main byproduct of this process is glycerol, which constitutes ca. 10% in weight of the produced raw biodiesel (Hoogendoorn and van Kasteren, 2010; Saikia et al., 2022). To promote a “zero waste” economy, the valorization of glycerol gained increasing interest during recent years. To this end, converting glycerol to solketal represents an appealing possibility. This product can be employed as a flavoring agent, a medicinal excipient or as a fuel additive, allowing to eventually carry out a circular production process (García et al., 2008; Saikia et al., 2022; Zhou et al., 2008). Solketal is selectively produced through the acid-catalyzed acetalization of glycerol with acetone (Saikia et al., 2022). Several homogeneous catalysts such as sulfuric acid, hydrochloric acid and p-toluenesulfonic acid have been employed in the past for solketal production (Corrêa et al., 2021; Talebian-Kiakalaieh et al., 2018). Their use presents several drawbacks, such as difficulties in separation and corrosion of reactors (Wang et al., 2024), which can be overcome by employing heterogeneous acid catalysts. Among the most promising solid catalysts reported in the literature for converting glycerol to solketal are metal-containing mesoporous silicates, zeolites, and metal organic frameworks (MOFs) (Dias et al., 2024). These materials usually incorporate transition metals into their structure, playing a crucial role in providing the acidity required to achieve high catalytic efficiency in the ketalization of glycerol. For instance, it was recently reported that silica-based materials containing trivalent or tetravalent metal cations, such as Ga(III)- (Soumoy et al., 2025), Sn(IV)- (Bivona et al., 2019), Zr(IV)- (Li et al., 2012) and Hf(IV)-doped silica nanostructures (Soumoy et al., 2022), were efficient catalysts towards the target reaction, giving promising productivity values per hour. It was recently shown by Sharma et al. (2025) that the presence of rhodium metal centers and an organic acid group like sulfonic acids on the same solid causes a synergistic combination which improves the performance of the catalyst.

In this scenario, porous metal phosphonate-/phosphates (PMPs) (Phosphonates, 2020) recently emerged as high-potential tunable acid catalysts which could be employed for different catalytic applications, including the conversion of glycerol to solketal. Combining the mechanical stability of an inorganic material and the structural properties of the organic acid groups allowed to obtain stable and highly efficient catalysts. PMPs are a class of materials that integrate di-topic organophosphonic acid linkers between rigid inorganic layers to form semi-organized frameworks in two or three dimensions, and these organic linkers tune the hydrophilic/hydrophobic balance by providing hydrophobic aromatic moieties, a factor that may influence acid-site accessibility and catalytic performance (Calogero et al., 2011; Lanari et al., 2011; Lv et al., 2021; Ma et al., 2011). In addition, the incorporation of spacers (i.e. phosphoric acid, monotopic phosphonic acids) not only enhances structural tunability and porosity but also provides a route to tailor the functional properties and active site distribution (Li et al., 2019). Inter-particle mesoporosity may be promoted by adjusting the spacer/linker ratio (Phosphonates, 2020). When high-valency metals are introduced (e.g. Ti(IV), Zr (IV), Sn(IV)), these materials feature hydrolytically strong M(IV)–O–P bonds that offer remarkable chemical and thermal stability, enabling them to endure harsh conditions (e.g. high acidic media below pH 1, elevated temperatures) commonly used in separation processes and catalysis (Pawlak et al., 2022; Silbernagel et al., 2016). Their structural versatility and chemical stability, guided by the choice of the M(IV) center, in combination with the diversity of functionalities in the hydrocarbon group of the organo-phosphonates and spacer/linker ratios, make PMPs particularly attractive for acid-catalyzed transformations such as the conversion of glycerol to solketal (Corrêa et al., 2021; Fatimah et al., 2019).

In this context, Zirconium (IV) is employed as the inorganic backbone and combined with rigid organic linkers, such as 1,4-Phenylenediphosphonic acid [PhDPA], which can covalently bridge zirconium centers to form semi-organized layered hybrid frameworks with increased porosity and tunable surface functionalities (Pawlak et al., 2022). Concurrently, phosphoric acid [oPA] can be incorporated as an inorganic spacer, intercalating between zirconia–linker layers (Silbernagel et al., 2016). The role of the spacer is crucial: it regulates interlayer ordering, it enhances porosity and it maintains the stability of the layered architecture (Díaz et al., 2007; Donnadio et al., 2024). Moreover, the presence of additional Brønsted acidic sites is a key factor in facilitating the acetalization of glycerol. Zirconium (IV) layered phosphonate–phosphates were reported by Silbernagel et al., where their synthesis, structural characterization, and ion-exchange properties were described (Silbernagel et al., 2016).

In this work, a more in-depth understanding of the structure–function relationship of PMPs is developed for the specific application of glycerol to solketal conversion. Special attention is given to the introduction of phosphate (spacers) as a source of Brønsted acidity, enabling to study the impact of this specific modification on the catalytic efficiency. A detailed understanding of the quantity and role of the Brønsted acidic sites and their catalytic relevance is obtained through a comprehensive approach involving Hammett titrations, solid-state Phosphorus-31 Nuclear Magnetic Resonance spectroscopy (^31^P-NMR), ammonia Temperature-Programmed Desorption (NH_3_-TPD) and X-ray Photoelectron Spectroscopy (XPS). Stability experiments were performed at elevated temperatures (up to 800 °C) to investigate the thermal stability of Zr(IV)-based PMPs. Moreover, the structural and acidic properties of the synthesized catalysts were correlated to their catalytic activity.

Studying these Zirconium (IV) phosphonate-phosphate hybrid materials not only deepens the understanding of structure-activity relationships in solid acid catalysts but also provides a promising pathway towards further design of efficient materials for biomass valorization and green chemistry applications (Okuhara, 2002).

## 2 Materials and methods

### 2.1 Material synthesis

All chemicals were used as received without any additional purification. 1,4-Phenylenediphosphonic acid (PhDPA,>98%) was purchased from TCI Chemicals (Tokyo, Japan). Zirconyl chloride octahydrate (ZrOCl_2_·8H_2_O), Phosphoric acid (oPA,>85%), *n*-butylamine (>99.45%), Methyl red (ACS reagent) and Petroleum ether (ACS reagent) were purchased from Sigma-Aldrich (Saint Louis, United States). The 120 mL Teflon vessels (model 4,748) used in the hydrothermal experiments were purchased from Parr (Moline, United States). Acid digestions were performed in Suprapur® 40% HF obtained from Merck (Darmstadt, Germany) and Instra-Analyzed® 69% HNO_3_ obtained from J.T. Baker (Phillipsburg, United States). Ultrapure (UP) water (18 MΩ cm) was obtained using an Arium Pro System (Sartorius, Göttingen, Germany). Glycerol (≥99,7%), acetone (≥99,8%), HCl (2 M) were purchased from Carl Roth.

Zirconium phosphonate-phosphate samples were synthesized based on a methodology established by Silbernagel et al. (2016) by dissolving 2.14 mmol of 1,4-phenylenediphosphonic acid (C_6_H_4_(PO_3_H_2_)_2_, PhDPA) in 15.70 mL of ultrapure water. For catalyst ‘Zr-PhDPA-oPA’, 17.15 mmol of phosphoric acid (H_3_PO_4_, oPA) was added to the reaction mixture to integrate phosphate groups into the framework. This resulted in a molar ratio of phosphonates to phosphates of 1:8 in the reactant solution. In contrast, for Catalyst ‘Zr-PhDPA’, no oPA was added, targeting a phosphate-free material. However, the amount of phosphonate incorporated remained identical to that in catalyst ‘Zr-PhDPA-oPA’, ensuring a consistent phosphonate concentration in solution for both samples. Next, 21.44 mL (10.72 mmol) of a 0.5 M zirconyl chloride octahydrate (ZrOCl_2_·8H_2_O) solution was added dropwise in both cases under constant stirring. Upon addition of the zirconyl chloride solution, immediate precipitation was observed for both samples. The reaction mixture was transferred into a Teflon-lined pressure vessel and heated at 120 °C for 4 days. After cooling down to room temperature, the precipitate was collected by vacuum filtration and thoroughly washed with UP water to remove residual reactants. The filtered solid was dried overnight in a vacuum oven at 60 °C and ground into a fine, white powder for further analysis.

### 2.2 Compositional analysis

A microwave acid digestion of the hybrid zirconia powders was performed in a Milestone Ethos reactor (Sorisole, Italy) by adding 5–20 mg of sample to 7.5 mL of HNO_3_ and 2.5 mL of HF in a Teflon vessel and ramping the temperature to 220 °C in 20 min, at which the sample was held isothermally for another 15 min. The resulting mixtures were allowed to cool to room temperature before being diluted with UP water in 50 mL polypropylene flasks. The mixtures were then analyzed on an Optima 8300 DV ICP-AES instrument (PerkinElmer, Waltham, United States) in axial view mode. Instrument calibration was done using the Merck zirconium standard reference Certipur® and VWR Chemical (Radnor, United States) Phosphorus Plasma Emission Standard dilutions between 0.01 and 100 mg/L.

Solid-state ^31^P magic angle spinning (MAS) NMR spectra were acquired at ambient temperature on an Agilent VNMRS DirectDrive 400 MHz (9.4 T wide bore magnet) spectrometer (Santa Clara, United States) equipped with a T3HX 3.2 mm probe. MAS was performed at 15 kHz using 3.2 mm zirconia rotors (22 µL rotors). The ^31^P resonance frequency was 161.98 MHz. Acquisition parameters used were a spectral width of 60 kHz, a 90° pulse length of 3.9 µs, an acquisition time of 15 ms, a recycle delay time of 30 s, and 800 accumulations.

### 2.3 Structural and surface analysis

N_2_ physisorption measurements were carried out at 77 K using a Tristar II 3020 surface area analyzer (Micromeritics, Norcross, United States). Prior to a measurement, the sample was degassed under nitrogen flow at 150 °C for 16 h to remove any residual moisture and other adsorbed components. The specific surface area (S_BET_) was estimated using the Brunauer-Emmett-Teller (BET) theory.

Ambient Powder X-ray Diffraction (PXRD) was collected using a Malvern Panalytical Empyrean diffractometer (Malvern, UK), using a PIXcel3D solid-state detector in scanning mode and a Cu anode (Cu Kα1 = 1.5406 Å; Cu Kα2 = 1.5444 Å) operating at 40 mA and 45 kV. The spectra were collected in transmission mode over a 2θ range of 1.2° < 2θ < 45° with a step size of 0.0131° and a counting time of 100 s.

The temperature-dependent *in situ* XRD measurements were carried out using a Malvern Panalytical Empyrean diffractometer equipped with non-monochromated Cu radiation (Cu K_α1_ 1.540,598 Å; Cu K_α2_: 1.544,426 Å), Detector PIXcel 3D 1 × 1 (solid state pixel detector) using the Bragg-Brentano geometry (reflection). The incident beam slits consisted of a Soller slit (0.02 rad) and a divergence slit (1/4°); whereas on the diffracted beam side, a Soller slit (0.02 rad) was used. A diffractogram was collected at different temperatures: 60 °C, 230 °C, 300 °C and 400 °C. Once the target temperature was reached, the diffractograms were collected after an equilibration time of 1 h. Additional *ex situ* diffractograms were recorded after thermal treatments at 600 °C and 800 °C to assess high-temperature stability.

The morphology of the materials was assessed via transmission electron microscopy (TEM), carried out using a Philips Tecnai 10 device operating at 80 kV. The samples were prepared by dispersing the solids in 2 mL of ethanol, sonicating the mixture, and drop casting onto a Formvar-coated copper grid. Additionally, scanning electron microscopy (SEM) was performed using a FEI Quanta 200F field emission gun microscope (FEG-SEM). Chemical analysis was performed using a commercial electron spectrometer (PHI-5600LS) equipped with monochromatized Al-K_α_ radiation (1,486.6 eV).

The Hammett titration method with *n*-butylamine was used to determine the Brønsted surface acidity of the catalyst (Benesi, 1957). The catalyst samples (100 mg each) were pre-dried at 60 °C in a vacuum oven to eliminate the influence of adsorbed water on the acidity measurements. After drying, each sample was suspended in 3 mL of petroleum ether, an apolar solvent used to titrate the acidic groups without dissolving the material. Approximately 100 µL of methyl red solution (0.1 g/L in benzene) was then added as a Hammett indicator. The suspension was stirred for 10 min to ensure proper mixing before *n*-butylamine, the titrant selected for its compatibility with the Hammett acidity function scale, was gradually added. The equivalence point, indicated by the color change, was recorded to determine the surface acidity of the catalyst. Each sample was analyzed in triplicate, and results are reported with error bars denoting one standard deviation.

In addition, the total acidity of materials was determined using the ammonia temperature-programmed desorption (NH_3_-TPD) method, conducted on a Hiden Catlab gas analyzer equipped with a QGA Hiden quadrupole mass spectrometer. Ammonia TPD was performed to assess the acidity of the samples, starting with the degassing of a catalyst sample (20–30 mg) at 150 °C for 1 h under a 30 mL/min Ar flow. NH_3_ adsorption was then carried out at 60 °C for 1 h using a gas mixture of 95% Ar and 5% NH_3_-He, each supplied at a flow rate of 15 mL/min. Subsequently, NH_3_ desorption was monitored under a 30 mL/min Ar flow as the temperature increased from 60 °C to 550 °C at a rate of 10 °C/min. The total acidity was quantified by integrating the area under the peaks in the desorbed molecules per second *versus* the desorption temperature plot.

XPS analysis was performed on a PHI VersaProbe III (ULVAC-PHI, Japan). During the measurements, an Al K_α_ monochromatic X-ray source (25 W, 15 kV) and dual-beam charge neutralization (a 1 V electron beam and a 7 V Ar^+^ ion beam) were applied. High-resolution scans of the Zr 3d, Zr 3p, C 1s, N 1s, O 1s, and P 2p photoelectron peaks were recorded from a spot diameter of 100 μm using a pass energy of 26 eV and a step size of 0.1 eV. Measurements were performed with a take-off angle of 45° with respect to the sample surface. The powders were applied on double-sided scotch tape, and the vacuum in the analysis chamber was approximately 9 × 10^−7^ Pa. The energy scale of the XPS spectra was calibrated relative to the binding energy of C=C in C 1s at 284.5 eV. Data analysis was performed with PHI MultiPak.

Thermogravimetric analysis (TGA) was performed using a TA Instruments Q500 apparatus (TA Instruments,United States). The temperature profile consisted of a heating ramp of 5 °C/min from room temperature (RT) to 800 °C. A dynamic dry air sample gas flow of 60 mL/min was implemented.

Thermogravimetric analysis coupled with mass spectrometry (TGA-MS) was performed using a TA Instruments Q5000 TGA system (TA Instruments,United States) coupled to a ThermoStar™ quadrupole mass spectrometer (Pfeiffer Vacuum, Germany). The temperature program consisted of a heating ramp of 5 °C/min from room temperature to 800 °C in 50 mL/min of dry air, providing an oxidative atmosphere. Evolved gas analysis was conducted via mass spectrometry, operating under high vacuum with the Pfeiffer vacuum system. The mass spectrometer scanned a mass-to-charge (m/z) range of 10–150 with a dwell time of 0.1 s per m/z.

### 2.4 Catalytic performance

#### 2.4.1 Catalytic conversion

0.92 g of glycerol (0.01 mol), 10 mg of catalyst and 2.32 g of acetone (0.04 mol) were added to a 10 mL round-bottom flask. The vessel was immersed in an oil bath at 50 °C, under reflux, stirring at 800 rpm. After 2 h, the reaction mixture was cooled down, followed by the addition of 3 mL of absolute ethanol. The mixture was sonicated for 1 min and then centrifuged at 4,500 rpm for 10 min 500 μL of the liquid was withdrawn and evaporated with a rotavapor under 50 mbar, 50 °C for 10 min 500 μL of DMSO-D6 was added to the flask and the solution was injected into an NMR tube. The products were quantified by integration of the signals of the secondary hydroxyl group of glycerol (doublet at 4.46 ppm), the solketal hydroxyl group (triplet at 4.75 ppm) and the byproduct (i.e., 2,2-dimethyl-1,3-dioxane-5-ol) hydroxyl group (doublet at 4.90 ppm). The conversion (see Equation 1), the yield (see Equation 2), and the selectivity (see Equation 3), were obtained as indicated in the formulas reported below:

$$Conversion=\frac{n_{glycerol\;}^{i}-n_{glycerol}^{f}}{n_{glycerol}^{i}}\cdot 100=\frac{n_{solketal}+n_{byproduct}}{n_{glycerol}^{f}+n_{solketal}+n_{byproduct}}\cdot 100$$ (1)

$$Yield=\frac{n_{solketal}}{n_{glycerol}^{f}+n_{solketal}+n_{byproduct}}\cdot 100$$ (2)

$$Selectivity=\frac{n_{solketal}}{\;n_{solketal}+n_{byproduct}}\cdot 100$$ (3)

where n^i^_glycerol_ is the initial number of moles of glycerol, n^f^_glycerol_ is the final number of moles of glycerol, n_solketal_ and n_byproduct_ are respectively the number of moles of product and byproduct.

#### 2.4.2 Leaching test

Catalyst Zr-PhDPA-oPA: 0.92 g of glycerol (0.01 mol), 10 mg of catalyst, 2.32 g of acetone (0.04 mol) and 600 μL of absolute ethanol were added to a round-bottom flask and it was immersed in an oil bath at 50 °C, stirring at 800 rpm under reflux. After 1 h, the reaction was stopped, and the mixture was filtered. 500 μL of the reaction mixture were recovered, evaporated at 50 mbar, 50 °C for 10 min and then analyzed via ^1^H-NMR after addition of 500 μL of DMSO-D6, while the rest of the liquid was left to react for another hour. 500 μL of the liquid were then withdrawn and evaporated with a rotavapor under 50 mbar, 50 °C for 10 min 500 μL of DMSO-D6 were added to the flask, injected in an NMR tube and analyzed as described in the previous section.

Catalyst Zr-PhDPA: 14.72 g of glycerol (0.16 mol), 10 mg of catalyst and 37.12 g of acetone (0.64 mol) and 9.6 mL of absolute ethanol were added in a round-bottom flask. After 20 min, the reaction was stopped, and the mixture was filtered. A similar procedure as described above was employed to analyze the catalytic results of Catalyst Zr-PhDPA.

#### 2.4.3 Recycling

The first cycle was carried out using 2.3 g of glycerol (0.025 mol), 5.8 g of acetone (0.1 mol), 25 mg of catalyst, stirring at 800 rpm, at 50 °C for 2 h. The same procedure as reported in section 2.4.1 was followed, but at the end of the reaction, the catalyst was recovered via centrifugation, dispersed in 3 mL of HCl 2 M and stirred for 1 h at 25 °C. The material was then washed with water until the pH of the supernatant became neutral, subsequently filtered out and dried in a vacuum oven at 60 °C for 16 h before carrying out the following cycle. The quantities of reactants used were scaled down at each cycle depending on the amount of recovered catalyst. This procedure was repeated for four cycles. After the 4^th^ cycle, the acid treatment was performed under the same conditions, but the catalyst was stirred in HCl at a temperature of 50 °C.

## 3 Results and discussion

### 3.1 Composition and structural properties of the obtained hybrid porous zirconium (IV) phosphonate-/phosphate

**FIGURE 1:**
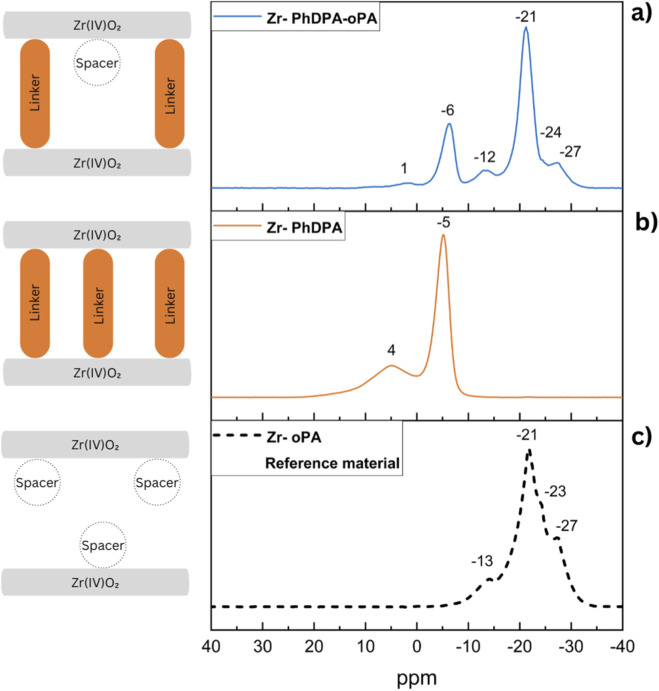
Solid-state ^31^P MAS NMR spectra of ‘Zr-PhDPA-oPA **(a)**, and ‘Zr-PhDPA’ **(b)**, with ‘Zr-oPA’ **(c)** as a reference material. The drawings are merely representative and not intended to depict the actual structures of the materials.

The composition of the synthesized materials, determined by ICP-AES, indicate that zirconium and phosphorus are incorporated in proportions closely matching the initial feed ratios, confirming the efficient reaction between the zirconyl chloride precursor and the phosphate and/or phosphonate reagents (Supplementary Table S1). In addition, no residual P and Zr could be detected in the supernatant after hydrothermal treatment and in the washing water. This consistency reflects the high efficiency of the synthesis process, which ensures that the expected stoichiometry of the Zirconium (IV) phosphate-phosphonate framework is attained (Nakamura et al., 2021). Subsequently, the phosphonate/phosphate ratio, their corresponding local chemical environment(s) and binding mode(s) are elucidated by solid-state ^31^P-MAS-NMR. This technique is particularly effective in revealing structural properties and binding modes of the phosphonates and phosphates, especially in amorphous or semi-ordered systems, where conventional diffraction methods may provide limited information (Bakhmutov et al., 2022). For ‘Zr-PhDPA-oPA’, multiple resonance signals associated with both phosphonate and phosphate moieties are observed. In ‘Zr-PhDPA’, the latter environment is not present, as expected (Supplementary Figure S1). The signal at −6 ppm represents the dominant binding mode of the phosphonate linker, indicating a successful bridging of PhDPA between two neighboring inorganic layers via anchoring of both -PO_3_H_2_ groups and thereby creating an interlayer spacing imposed by the size of the rigid phenyl group. Similarly, a second major contribution observed for the phosphate spacer at −21 ppm is likely associated with the (ZrO)_3_POH or (ZrO)_3_P = O binding mode, reflecting its interaction within the zirconium framework (Mutin et al., 2005; Ou et al., 2020; Silbernagel et al., 2016). In addition to the dominant signals associated with PhDPA and oPA, contributions at 1, -13, −24, and −27 ppm are observed, suggesting the presence of multiple binding modes for both species. The phosphonate-to-phosphate molar ratio in the synthesis is maintained at 1:8, hence the expected integration ratio in the ^31^P-MAS-NMR spectrum should be 1:4, as each PhDPA contains two phosphorus atoms. The quantitative integration of the spectral regions which are conventionally assigned to phosphonate (+5 ppm to −10 ppm) and phosphate (−15 ppm to −28 ppm) environments deviates from this expected 1:4 ratio (Supplementary Figure S1). However, ICP-AES results confirm that neither phosphorus nor zirconium is lost during the synthesis and washing steps (Silbernagel et al., 2016). In order to look at the partial overlap in detail, a reference material composed of zirconium with only a phosphate spacer ‘Zr-oPA’ was specifically synthesized. This system provides a simplified model that aids in understanding the binding modes of the phosphate spacer to the zirconium surface (Figure 1). In ‘Zr-PhDPA’, the signal at 4 ppm shows a clear upfield shift to the corresponding peak at 1 ppm in ‘Zr-PhDPA-oPA’, indicating a change in the local phosphonate environment that could originate from the introduction of neighboring phosphate groups (spacers). This creation ofing additional non-covalent interactions or changes in the coordination geometry of the phosphonate group may lead to an increased structural disorder. Additionally, the main phosphonate peak, observed at −5 ppm in ‘Zr-PhDPA’, is shifted upfield to −6 ppm in Zr-PhDPA-oPA’. When compared with the reference material ‘Zr-oPA’, which shows phosphate resonances in a region −7 to −32 ppm, it becomes evident that spectral overlap occurs in Zr-PhDPA-oPA, and that an underestimation of the phosphate occurs. These overlapping resonances complicate the deconvolution and accurate integration of the phosphate and phosphonate contributions, as shown in the integration analysis (Supplementary Figure S1). Hence, the spectral convolution affects the reliability of quantitative phosphate-to-phosphonate ratio determinations based solely on direct integration (Bakhmutov et al., 2022; Silbernagel et al., 2016).

The influence of the orthophosphate spacer on the porosity and specific surface area is illustrated by the N_2_ physisorption isotherms (Supplementary Figure S2), with ‘Zr-PhDPA-oPA’ exhibiting a specific surface area (S_BET_) of 410 m^2^/g. The sharp increase in the adsorption isotherms at low relative pressure (ca. 0.02 p/p°) indicates the presence of micropores, potentially formed by the spacers creating voids in the condensed organophosphate layer formed by the linkers. The slow uptake at higher relative pressures indicates the presence of disordered mesopores, facilitating improved accessibility and diffusion pathways (Clearfield, 2008; Silbernagel et al., 2016). In the absence of oPA (e.g. in the case of Zr-PhDPA), a reduced surface area of 250 m^2^/g was detected, as the loss of spacer-induced microporosity results in densification of the layered structure due to the closer vicinity of the phenyl rings of the PhDPA linker. The nitrogen adsorption–desorption isotherm exhibits a distinct hysteresis loop, giving insights on the mesoporous nature of the material. The open hysteresis observed in the desorption branch can be ascribed to capillary condensation and delayed evaporation processes, which are typically associated with the presence of ink-bottle-shaped pores and interparticle voids. Similar behavior has been associated with disordered, house-of-cards-like arrangements of plate-like particles, as reported by Sanchez-Cantu et al. (2010).

XRD analysis revealed no reflections assignable to monoclinic ZrO_2_ (COD ID 5000038) (Martin et al., 1993) or to crystalline α-Zr(HPO_4_)_2_·H_2_O (COD ID 1529538) (Clearfield and Stynes, 1964), indicating that the synthesized material does not correspond to either of these well-defined reference phases. Instead, the diffractogram is consistent with the formation of a semi-ordered (hybrid) structure. The four broad peaks in the diffractogram of ‘Zr-PhDPA-oPA’ (Supplementary Figure S3) indicate the nanocrystalline nature of the samples, consistent with the absence of long-range periodic order. The reflection at around 8.5° (2θ) corresponds to an average interlayer spacing of 10.4 Å, providing insight into the structural arrangement of the material. For ‘Zr-PhDPA’, this low-angle reflection is less pronounced, pointing to a reduction in interlayer ordering compared to ‘Zr-PhDPA-oPA’. This reduction in interlayer ordering can be attributed to the absence of phosphate groups in the structure (Alberti et al., 1992; Mutin et al., 2003; Silbernagel et al., 2016). Transmission electron microscopy (TEM) showed that ‘Zr-PhDPA-oPA’ contained sheet-like domains and regions with parallel contrast fringes, while ‘Zr-PhDPA’ appeared more granular, with predominantly spherical or irregularly shaped particles and without clear planar features (Supplementary Figure S4) (Ulusoy, 2023).

### 3.2 Acidity properties of the hybrid porous Zirconium (IV) phosphonate-phosphates and phosphonates

The accessibility and nature of acidic sites are closely correlated with the catalytic activity of the porous zirconium phosphonate–phosphate and zirconium phosphonate. To elucidate these properties, Brønsted acidity was examined by n-butylamine titration with Hammett indicators coupled with ^31^P-MAS-NMR at different titration stages, while NH_3_-TPD was employed to assess the distribution and strength of the acidic sites. Together, these complementary techniques enabled a comprehensive evaluation of the quantity and characteristics of acidity in the samples (Sasca et al., 2016).

**FIGURE 2:**
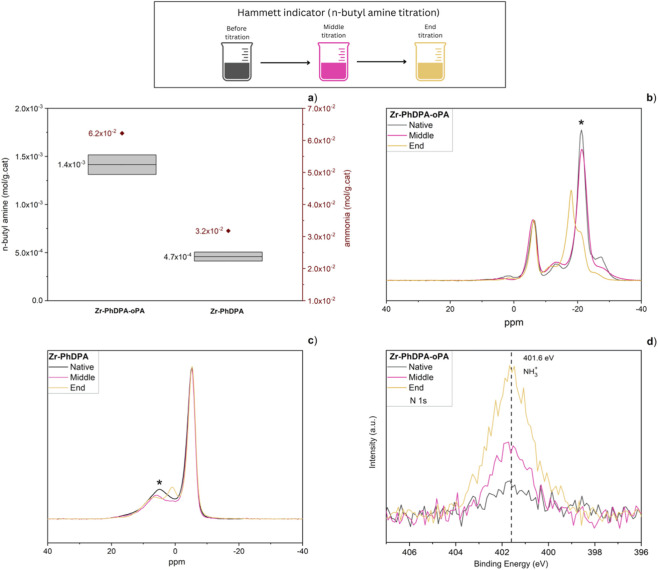
Acidity measurements using the Hammett indicator and ammonia TPD **(a)** ^31^P-MAS-NMR spectra of ‘Zr-PhDPA-oPA’ at three different stages of the titration **(b)** ^31^P-MAS-NMR spectra of ‘Zr-PhDPA’ at three different stages of the titration **(c)** X-ray photoelectron spectroscopy (XPS) analysis of ‘Zr-PhDPA-oPA’ at the three different stages of titration, focusing on the N 1s spectral region **(d)**.

The quantification of Brønsted acidic groups based on the Hammett indicator method (Figure 2a) revealed that ‘Zr-PhDPA-oPA’ exhibited 1.4 ± 0.1 × 10^−3^ mol/g·cat of acidic sites, which is approximately three times higher than that of ‘Zr-PhDPA’ (4.7 ± 1.1 × 10^−4^ mol/g·cat). The significantly higher acidity of ‘Zr-PhDPA-oPA’ can likely be attributed to the incorporation of additional phosphate (spacer) functionalities within the interlayers (Benesi, 1957; Liu et al., 2013). Additionally, NH_3_-TPD analysis was performed to identify the distribution and strength of the catalysts’ acidic sites. The TPD acidity profiles of ‘Zr-PhDPA-oPA’ and ‘Zr-PhDPA’ are reported in Supplementary Figure S5, highlighting significant differences in their acidic site distributions. Whereas ‘Zr-PhDPA-oPA’ exhibited both weak (i.e. ammonia desorption peak at 150 °C) and strong acidic sites (shoulder around 300 °C), ‘Zr-PhDPA’ displayed mainly weak acidic sites, which can be attributed to residual Zr-O-H functionalities or to uncoordinated P–OH groups. In addition, the quantification of the acidic sites in NH_3_-TPD is consistent with the above-described Hammett indicator approach, although higher absolute values are found, which could be associated with the larger kinetic diameter of the *n*-butylamine probe molecule, not reaching all acidic sites in the microporous structure, limiting its adsorption to mesoporous and external surface regions (Dědeček et al., 2012). The pronounced difference in acidity between the two catalysts is attributed to the availability of uncoordinated P-OH groups of the incorporated phosphates in ‘Zr-PhDPA-oPA’. To gain deeper insight into the individual contributions of the phosphate spacer and phosphonate linker to the total acidity of the porous zirconia phosphonate, ^31^P-MAS-NMR spectra were measured at three stages of the Hammett titration: before titration (native sample), after the addition of half the equivalent volume of titrant (i.e. at the middle-point), and at the equivalence point (i.e. end point), as shown in Figures 2b,c. Indeed, as illustrated in Figures 2b, the phosphate group is the dominant contributor in the titration process since the peak (−21 ppm) associated with this moiety is strongly broadened, and the peak shape alters significantly during the titration process. In contrast, the main peak of PhDPA (linker) remains unaltered during the titration (Vila, 1991; Zheng et al., 2011). Hence, it can be hypothesized that both P–OH functionalities of each phosphonate group in PhDPA are completely bonded with the inorganic layers, leaving little residual Brønsted acidity; it may also be the case that some P–OH groups remain uncoordinated but are not accessible to n-butylamine. In ‘Zr-PhDPA’, only minor changes are observed during the titration (Figures 2c). The signal at the higher chemical shift (4 ppm) shows a more noticeable change, suggesting association with a binding mode involving unbound P–OH groups (mono- and/or bidentate).

X-ray Photoelectron Spectroscopy (XPS) measurements were conducted on the same three samples of the ‘Zr-PhDPA-oPA’ catalyst at distinct stages of the Hammett titration, The surface elemental ratio of phosphorus to zirconium in the native sample was 1.91 ± 0.04 (Supplementary Table S2), closely aligning with the bulk value of 1.69 ± 0.01 obtained from ICP-AES (Supplementary Table S1). This suggests that both PhDPA and oPA are nearly uniformly distributed throughout the material and remain accessible at the surface. As XPS can confirm proton transfer through binding energy shifts arising from acid–base interactions with n-butylamine (Ma et al., 2023; Stevens et al., 2010), the N 1s region of the XPS spectra was analyzed (Figures 2d). In the native sample, a slight increase in the background was observed, originating from traces of atmospheric contamination. As the titration progressed, an increase in a symmetrical peak at 401.6 eV was observed, which can be assigned to protonated–NH_3_^+^ groups, originating from a proton transfer from P-OH groups. Further evidence of a proton transfer during the titration is provided by the O 1s XPS spectra (Supplementary Figure S6a). The spectrum reveals a main peak at ∼531.2 eV, attributed to oxygen species in Zr–O–P and Zr–OH environments, along with a shoulder at higher binding energy (∼533 eV), assigned to P–OH groups (Fernández et al., 2019; Lin and Yuan, 2014). As the titration progresses, a gradual decrease in the relative intensity of the P–OH component is observed, consistent with the deprotonation of surface P–OH sites and supporting the conclusion that these phosphoric acidic hydroxyl groups are involved in proton donation to n-butylamine. This trend is further evidenced by the fitted O 1s spectra of the native and end titration stages (Supplementary Figures S6b,c), where a noticeable reduction in the P–OH peak (∼532.4–533.5 eV) is observed after titration, confirming the progressive loss of protonated oxygen species. These changes, together with the N 1s data, confirm that proton transfer occurs at the solid–liquid interface during titration, and that the acidic sites remain chemically accessible throughout the process. As shown in Supplementary Figures S6d,e, the P 2p and Zr 3d spectra remain essentially unchanged throughout the titration process, indicating that the local chemical environments of phosphorus and zirconium are not significantly affected, further evidencing that the titration primarily involves proton exchange at surface hydroxyl groups rather than changes to the inorganic framework. A similar set of experiments was conducted on the ‘Zr-PhDPA’ structure on the native sample and at the end of the titration. The surface elemental ratio of phosphorus to zirconium (P/Zr) in the native sample, as determined by XPS, was found to be 0.63 ± 0.08 (Supplementary Table S2), which is higher than the bulk value of 0.46 ± 0.003 obtained from ICP-AES (Supplementary Table S1). This difference indicates a relative enrichment of the phosphonate linker at the surface. As shown in Supplementary Figure S6f,g, the N 1s and O 1s XPS spectra of ‘Zr-PhDPA’ display only minor changes between the native and titrated sample. These subtle variations are consistent with the lower amount of *n*-butylamine used during titration, as well as the minor changes observed in the ^31^P-NMR spectra. This suggests limited proton transfer and supports the conclusion that fewer accessible acidic sites are present in this material compared to ‘Zr-PhDPA-oPA’. Supplementary Figure S6h,i demonstrate that the P 2p and Zr 3d spectra indicate stable chemical environments during the titration.

### 3.3 Stability of the hybrid porous Zirconium (IV) phosphonate-phosphates

‘Zr-PhDPA-oPA’, which exhibits a higher Brønsted acidity than ‘Zr-PhDPA’, was identified as the more promising candidate for catalytic conversion, prompting further investigations to better understand its structure–performance relationship, as well as a detailed assessment of its stability, which is essential for the envisioned application. Although the catalytic reaction itself occurs at relatively mild conditions (up to 50 °C), the thermal stability analysis was extended up to 800 °C to comprehensively evaluate the material’s robustness and structural integrity. Evaluating the material’s thermal stability is essential not only to ensure reliable catalytic performances but also to guarantee accurate and reproducible characterization results. For example, surface area analysis and acidity measurements require pre-treatment steps such as degassing and drying at temperatures up to 150 °C (as described in the Materials and Methods section), which expose the material to more severe conditions than the catalytic reaction itself (Ou et al., 2020). In this context, the sample’s hygroscopic nature and its reversible water adsorption were also investigated, as both can significantly influence catalytic performance and material handling. After heating ‘Zr-PhDPA-oPA’ to 150 °C to remove all physically and chemically bound water, it was exposed to ambient air for 5 and 10 min, followed by reheating to 150 °C. Remarkably, the sample showed a weight loss of 11% after just 5 min and 14% after 10 min exposure, indicating rapid water uptake from the atmosphere (Supplementary Figure S7). The high hygroscopicity is attributed to the presence of phosphate moieties at the surface. To verify this, an identical experiment was carried out on ‘Zr-PhDPA’. Indeed, the absence of phosphate groups proves to be an important contributor to the water uptake: this sample showed a mass loss of 7.5% between room temperature and 150 °C, and a more limited rehydration was evidenced by the 9.4% mass loss after 10 min of air exposure. For comparison, dehydrated ZrO_2_ also exhibited a mass loss of 7.5% under identical heating conditions (Supplementary Figure S8), confirming that the phosphonate linkers in ‘Zr-PhDPA’ do not significantly contribute to moisture uptake. This reversible water adsorption behavior of ‘Zr-PhDPA-oPA’ is consistent with the hydrophilic nature of phosphates and surface Zr–OH functionalities within the framework (Donnadio et al., 2024; Lanari et al., 2011).

**FIGURE 3:**
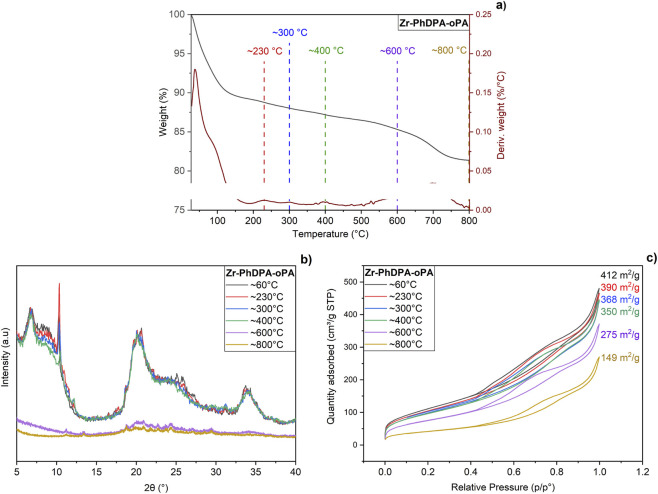
TGA results of ‘Zr-PhDPA-oPA’ **(a)** XRD analyses of ‘Zr-PhDPA-oPA’ after thermal treatment **(b)** BET analyses of ‘Zr-PhDPA-oPA’ after thermal treatment **(c)**.

To complement these findings and gain a more comprehensive understanding of ‘Zr-PhDPA-oPA’s thermal behavior beyond moisture uptake, detailed thermogravimetric and mass spectrometric analyses were performed. Apart from the initial (reversible) weight loss due to the removal of adsorbed moisture up to 150 °C, additional decomposition steps were observed at approximately 230 °C, 300 °C, 400 °C, 600 °C, and 800 °C (Figure 3a, DTG maxima). The TG-MS profile of ‘Zr-PhDPA-oPA’ (Supplementary Figure S9) reveals distinct decomposition stages. Notably, the decomposition steps observed at ∼230 °C, ∼300 °C, and ∼400 °C are all accompanied by a clear increase in the m/z = 18 signal, indicating water release due to hetero-condensation reactions, involving the remaining dangling hydroxyl groups from phosphate or phosphonate units, and the Zr–OH functionalities (Matta et al., 1999). At approximately 600 °C, another significant weight loss is detected, again accompanied by a pronounced m/e = 18 signal, suggesting continued water evolution—likely from deeper framework condensation processes or the final elimination of residual hydroxyl functionalities. This aligns with findings reported by Alvarez-Torrellas et al. for sulfated zirconia materials (Liu et al., 2016). The final mass loss near 800 °C, marked by a rise in the m/e = 44 signal, is attributed to CO_2_ release from the decomposition of remaining organic linkers and partial structural reorganization; no signals related to P-containing species were detected (Silva Filho et al., 2020). Given that all required thermal treatments for catalyst characterization and catalytic testing are conducted below , it can be concluded that only physisorbed moisture is removed under these conditions, with no alteration to the material’s structural o150 °Cr chemical integrity. The XRD patterns indicate structural integrity up to ∼400 °C, beyond which a loss of crystallinity between 400 °C and 600 °C indicates framework collapse. Notably, the disappearance of the interlayer reflection at ∼8.5° 2θ coincides with a significant loss of specific surface area from 412 m^2^/g to 275 m^2^/g, reinforcing the connection between structural collapse and diminished textural properties (Figures 3b,c). It can be anticipated that the collapse of the porous structure would have a direct impact on acidity, as the associated loss in surface area and accessible phosphate (spacer) sites is likely to significantly diminish the material’s effectiveness as an acid catalyst (Aboulayt et al., 2017; Wang et al., 2023). All observations, including the loss of crystallinity seen in XRD, the surface area reduction from BET analysis, and the decomposition trends identified by TGA and TG-MS, consistently confirm that thermal treatment beyond 400 °C disrupts the material’s framework integrity.

### 3.4 Catalytic tests

Catalytic tests for the conversion of glycerol into solketal were carried out using both ‘Zr-PhDPA-oPA’ and ‘Zr-PhDPA’. As emerges from Table 1, the two solids outperformed commercial zirconia, underlining the key role of the phosphonic and phosphoric moieties in conferring the required acidity to the materials (compare entries 1–3). The standard deviation associated with the catalytic results was calculated after performing four different tests under the same conditions with the same catalyst (see Supplementary Figure S10). The data reported in Supplementary Figure S10 highlight the high reproducibility of the catalytic tests.

**TABLE 1:** Catalytic activity of Catalyst ‘Zr-PhDPA-oPA and Zr-PhDPA’ in the conversion of glycerol to solketal. The catalysts were pre-treated at 60 °C in a vacuum oven overnight before carrying out the catalytic test. Entry 1: 0.01 mol of glycerol, 3.5 mg (3.5 × 10^−5^ mol) of commercial ZrO_2_. Entry 2 to 5: 0.01 mol of glycerol, 10 mg of catalyst. The TON was calculated as number of moles of solketal over the number of acid sites determined from NH_3_-TPD. Productivity was calculated as the mass of solketal over the mass of catalyst employed. The standard deviation of yield and selectivity values was 1. n.a.: not applicable.

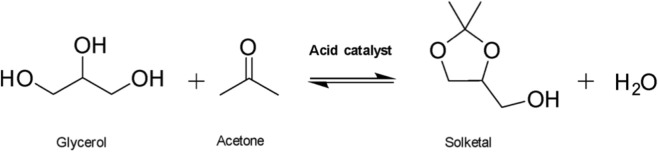								
Entry	Catalyst	Temp. (°C)	Time (h)	Glycerol: acetone	Yield (%)	Selectivity (%)	TON	Productivity
1	Commercial ZrO_2_	50	2	1 : 4	<2	n.a.	n.a.	n.a.
2	Zr-PhDPA-oPA	50	2	1 : 4	27	83	4	36
3	Zr-PhDPA	50	2	1 : 4	56	>99	18	74
4	Zr-PhDPA-oPA	50	24	1 : 15	85	98	14	112
5	Zr-PhDPA-oPA	25	24	1 : 15	46	77	7	61

When testing the two catalysts in the same conditions, ‘Zr-PhDPA’ (containing the linker but not the spacer) performed better than ‘Zr-PhDPA-oPA’ (containing in its structure both linker and spacer) towards the production of solketal, with higher TON and productivity (see Table 1, entries 2 and 3). This result was unexpected since ‘Zr-PhDPA-oPA’ presented a higher specific surface area and a larger amount of acid active sites than Catalyst ‘Zr-PhDPA’, as emerged from the NH_3_-TPD analysis (see Supplementary Figure S5).

To shed more light on the stability of these solids and to better understanding their catalytic behavior, leaching tests were carried out. Due to the different catalytic activity of the two materials, leaching tests were conducted under distinct reaction conditions to ensure that the system remained sufficiently far from equilibrium conversion. Interestingly, Zr-PhDPA-oPA’ did not leach active sites, while ‘Zr-PhDPA’ did (see Supplementary Figure S11), proving that the spacer plays a crucial role in the stability of the materials. We hypothesized that ‘Zr-PhDPA’ underwent hydrolysis during the catalytic test, causing the release of phosphonic acids, which catalyzed the reaction in homogeneous phase. This hypothesis was confirmed by performing an ICP analysis on the solid before and after the leaching test, showing a decrease in the phosphorus content from 14% to 6% of the total mass of the catalyst. This could explain the “apparent” better performances of ‘Zr-PhDPA’, which acted as a homogeneous catalyst under the selected reaction conditions. Taking this into account, ‘Zr-PhDPA-oPA’ can be considered as the most promising solid. Interestingly, upon increasing the amount of acetone employed in the catalytic test (entry 4, Table 1), the performance of ‘Zr-PhDPA-oPA’ significantly improved, reaching a yield of 85% after 24 h. Even at room temperature (entry 5, Table 1), the excellent catalytic performance of ‘Zr-PhDPA-oPA’ was still maintained.

**FIGURE 4:**
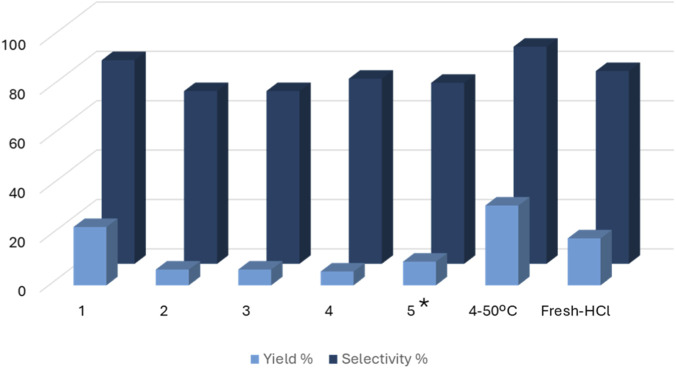
Recycling of Catalyst ‘Zr-PhDPA-oPA’. Conditions of the first tests: 50 °C, 2 h, 25 mg of catalyst, 0.025 mol of glycerol and 0.1 mol of acetone. The amount of reactants was scaled down cycle by cycle according to the amount of catalyst recovered. 4 °C–50 °C: results of the 4^th^ cycle of recycling of Catalyst ‘Zr-PhDPA-oPA’ after treatment at 50 °C with HCl 2 M. Fresh-HCl: test performed with the “fresh” catalyst after treatment with HCl 2 M.

**FIGURE 5:**
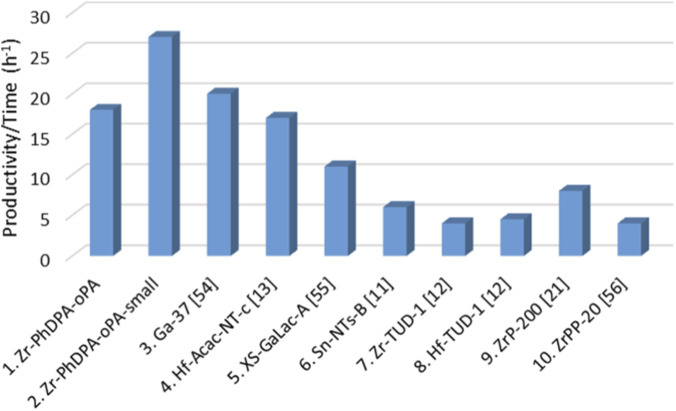
Comparison of ‘Zr-PhDPA-oPA’ and ‘Zr-PhDPA-oPA-small’ performances for the conversion of glycerol to solketal with some materials previously reported in literature. Conditions of the tests (columns 1–6): 50 °C, 0.01 mol of glycerol, 0.04 mol of acetone. Conditions of the tests (columns 7 and 8): 80 °C, 0.01 mol of glycerol, 0.01 mol of acetone. Conditions of the test (column 9): 50 °C, 0.02 mol of glycerol, 0.2 mol of acetone. Conditions of the test (column 10): 40 °C, 0.01 mol of glycerol, 0.04 mol of acetone (Bivona et al., 2019; Hussein et al., 2020; Li et al., 2012; Li et al., 2019; Li et al., 2020; Soumoy et al., 2022; Vivian et al., 2021)

To investigate the recyclability of the material, ‘Zr-PhDPA-oPA’ was reused for several consecutive cycles. Figure 4 illustrates a decrease of activity in the second cycle followed by stabilization in the two subsequent cycles. After each catalytic run, the catalyst was stirred in HCl 2 M for 1 h at room temperature. To remove the species strongly adsorbed onto the catalyst surface, between the fourth and the fifth cycle the temperature of the acid treatment was increased to 50 °C, improving the efficiency of the recycling procedure. A slight increase in yield was observed at the fifth cycle (see Figure 5, column 5*), confirming the importance of the acid treatment at 50 °C to reactivate the catalyst. To further explore this, the recycling of ‘Zr-PhDPA-oPA’ was also carried out by performing the acid treatment at 50 °C from the first cycle. This procedure allowed preserving the activity till the fourth cycle (see Figure 4, “4 °C–50 °C”, which corresponds to the fourth catalytic cycle of the aforementioned recycling procedure). However, a slight increase in the catalytic performance was also detected (compare cycle 1 and “4 °C–50 °C”). It should be mentioned that despite the high reproducibility of the catalytic tests (see Supplementary Figure S10), a slightly larger fluctuation of yield and selectivity was observed among the consecutive catalytic tests. This variation could be ascribed to the high hygroscopic nature of the solid (see Supplementary Figure S7), the sensitivity to acid treatment, and the variation in particle size (*vide infra*). To investigate if the acid treatment could cause a modification of the catalytic activity, the “fresh” catalyst was stirred in HCl 2 M for 1 h and it was tested in the conversion of glycerol to solketal under the same reaction conditions. The results of the test carried out with the pre-treated “fresh” catalyst are reported in Figure 4, “Fresh-HCl”, allowing to conclude that the acid treatment did not alter the performance of the material.

To comprehensively understand the catalytic activity of the ‘Zr-PhDPA-oPA’ and to further establish a structure–activity correlation, additional morphological characterization is provided. As illustrated in Supplementary SI-Supplementary Figure S12a,b, both SEM and TEM analyses revealed that the sample exhibited a heterogeneous particle size distribution, reflecting the material’s structural complexity. This diversity may have a significant influence on the catalyst’s performance. To understand if the particle size could influence the structural and textural features, and hence the catalytic activity, the ‘Zr-PhPDA-oPa’ samples were filtered (pore size 5–10 μm) to separate smaller particles from larger ones, creating two size fractions. The ‘Zr-PhDPA-oPA-small’ fraction was compared with the unfractionated sample via TEM (Supplementary SI-Supplementary Figure S12c,d). From this analysis, it emerged that the aggregates of ‘Zr-PhDPA-oPA’ were larger than the ones of ‘Zr-PhDPA-oPA’-small (compare Supplementary Figure S12c,d) and more dispersed.

To investigate whether these morphological differences were accompanied by variations in chemical composition, particularly in the distribution of phosphate and phosphonate moieties, ^31^P-MAS-NMR analysis was performed on the smaller-particle fraction (‘Zr-PhDPA-oPA-small’). The spectrum showed that the integrated area ratio of phosphate to phosphonate groups was 3.9 in ‘Zr-PhDPA-oPA-small’, compared to a lower ratio of 3.1 in the larger-particle fraction (Supplementary Figure S13). Although it was established previously that these ratios should only be interpreted relatively due to partial signal overlap (which seems to be less for ‘Zr-PhDPA-oPA-small’ as compared to ‘Zr-PhDPA-oPA’), it suggests that the smaller particles are relatively richer in catalytically active phosphate groups, which could account for differences in acidic behavior and potential catalytic performance (Smith et al., 2015).

Both ‘Zr-PhDPA-oPA’ and ‘Zr-PhDPA-oPA-small’ were tested as catalysts for the conversion of glycerol to solketal. ‘Zr-PhDPA-oPA-small’ displayed better catalytic performances (TON = 7 and productivity = 54) than ‘Zr-PhDPA-oPA’, confirming the presence of a higher phosphate/phosphonate ratio plays a key role in enhancing the activity of the solids.

The catalytic performances of ‘Zr-PhDPA-oPA’ and ‘Zr-PhDPA-oPA-small’ were included in the comparison of catalytic output parameters under the same conditions (Figure 5, Supplementary Table S3). To properly relate the catalytic results of different materials, the productivity overtime was calculated for each solid. ‘Zr-PhDPA-oPA’ and ‘Zr-PhDPA-oPA-small’ revealed to be promising heterogeneous catalysts for the synthesis of solketal, displaying a productivity/time equal to 18 h^-1^ and 27 h^-1^ respectively (Figure 5; Supplementary Table S3, Entry 1 and 2). These values were close to the ones shown in Supplementary Table S3, calculated for Ga-SiO_2_ catalysts (entries 3 and 5) and Hf-SiO_2_ nanotubes (entry 4), which presented outstanding performances compared to other materials recently reported in literature (Hussein et al., 2020; Soumoy et al., 2022; Vivian et al., 2021). ‘Zr-PhDPA-oPA’ and ’Zr-PhDPA-oPA-small’ display better performance than other silica-based materials embedding Sn, Zr and Hf in their structure (Bivona et al., 2019; Li et al., 2012; Soumoy et al., 2022) (Supplementary Table S3, entries 6–8). Moreover, the catalytic activity of hybrid materials containing Zr and phosphate/phosphonate moieties (Supplementary Table S3, entries 9 and 10) (Li et al., 2019; Li et al., 2020) was compared to the one of ‘Zr-PhDPA-Opa’ and ‘Zr-PhDPA-oPA-small’, which presented higher productivity/time values, evidencing once again their excellent activity towards the target reaction.

## 4 Conclusion

In this study, layered zirconium phosphonate-phosphate materials were successfully synthesized and evaluated as recyclable heterogeneous catalysts for the ketalization of glycerol to solketal. The layered materials were synthesized with precise control over the incorporation of the phosphonate linker and the phosphate spacer. Subsequently, the acidity was characterized using Hammett indicators, supported by solid-state NMR, XPS, and ammonia TPD analysis, with both Hammett and TPD measurements confirming the essential role of phosphate incorporation on the acidic properties of the materials. Additionally, the linker material largely serves the purpose of enhancing the porosity through the creation of layered structures. Thermal stability of the studied compounds was evaluated in depth, showing phenomena at lower temperatures such as reversible water adsorption and desorption. Hetero-condensation might occur in the low-T region above 200 °C, whereas structural degradation and decomposition of the structure only occur at extremely high temperatures beyond the scope of catalytic usage. Catalytic tests for the conversion of glycerol to solketal demonstrated good correlation to the aforementioned role of the phosphate spacer as ‘active’ acidic moiety, achieving a glycerol conversion of 85%, a selectivity to solketal of 98%, and a recyclability maintained over 5 cycles with the sample presenting both linker and spacer, confirming the potential of these materials as stable and efficient heterogeneous catalysts for sustainable glycerol valorization. Future work could focus on fine-tuning the particle size distribution to further enhance reproducibility, while the tunable nature of these layered materials offers promising opportunities for broader application in acid-catalyzed transformations and sustainable catalytic processes.

## Data Availability

The original contributions presented in the study are included in the article/Supplementary Material, further inquiries can be directed to the corresponding authors.
