# Supplementary figures and images for: Zirconium (IV) layered phosphonate-phosphate as catalysts for the valorization of glycerol
Source: Front Chem. 2026 Jan 12;13:1735925. doi: 10.3389/fchem.2025.1735925 (PMC12833269; doi:10.3389/fchem.2025.1735925)

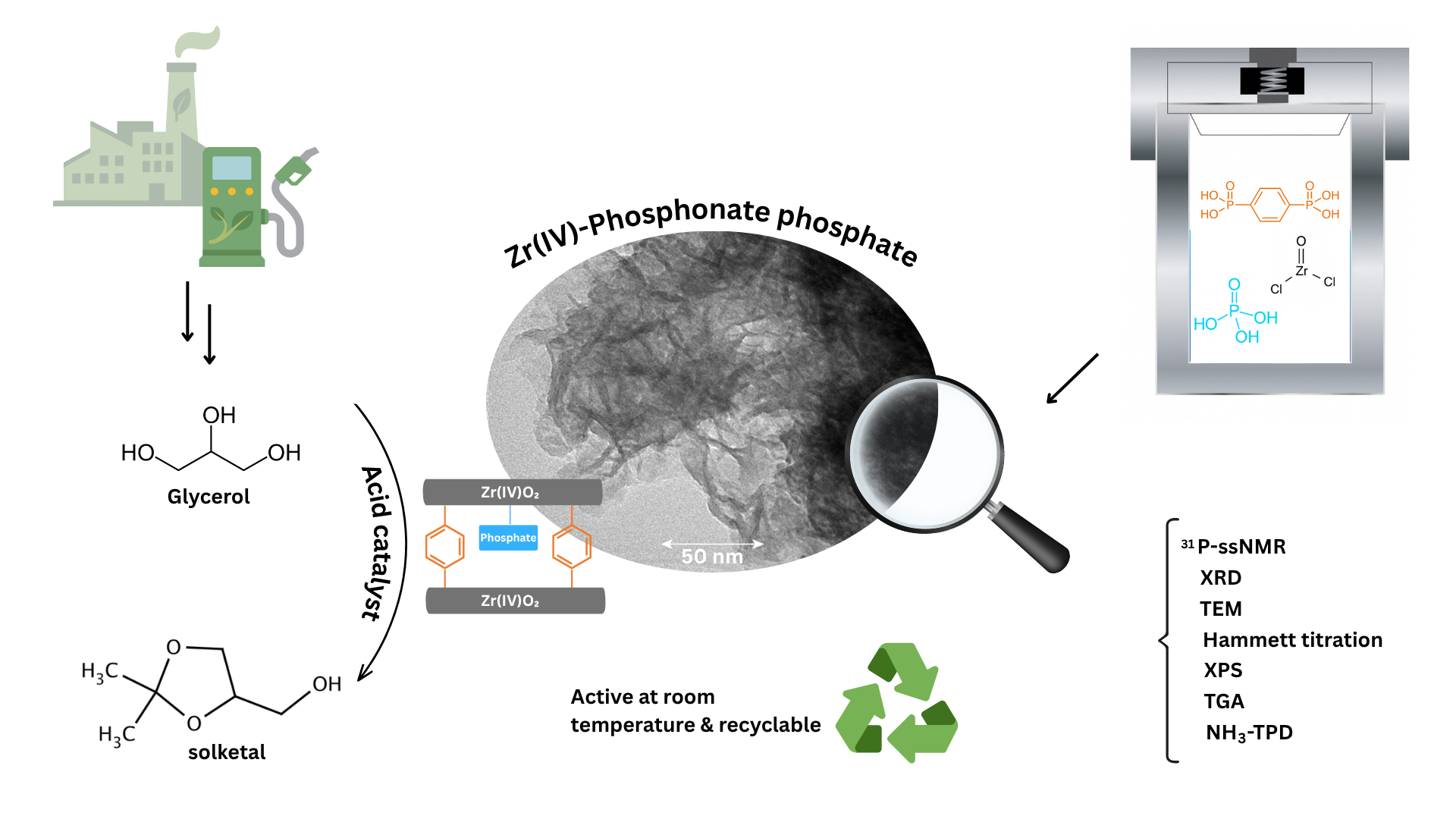

Supplement: Supplementary file 1 [file Image1.png]
