# Supplementary material for: Zirconium (IV) layered phosphonate-phosphate as catalysts for the valorization of glycerol
Source: Front Chem. 2026 Jan 12;13:1735925. doi: 10.3389/fchem.2025.1735925 (PMC12833269; doi:10.3389/fchem.2025.1735925)
Supplement: Supplementary file 2 [file DataSheet1.docx]

**Zirconium (IV) layered phosphonate-phosphate as catalysts for the valorization of glycerol**

Nahal Ghanemnia^1,†^, Martina Saitta^2,3,†^, Elien Derveaux^1,4^, Richa Tomer^3,6^

Nick Gys^5,6^, Tom Hauffman,^5^ Peter Adriaensens^1,4^, Sophie Hermans*^,3^, Carmela Aprile*^,2^, Wouter Marchal*^,1^

^1^ UHasselt, Institute for Materials Research (IMO-IMOMEC), Analytical and circular Chemistry (ACC), Agoralaan, 3590 Diepenbeek, Belgium

^2^ Laboratory of Applied Materials Chemistry, Unit of Nanomaterials Chemistry (UCNANO), Namur Institute of Structured Matter (NISM), Department of Chemistry, University of Namur, Namur, Belgium

^3^ Institute of Condensed Matter and Nanosciences (IMCN), Université catholique de Louvain (UCLouvain), Louvain-la-Neuve, Belgium

^4^ UHasselt, Institute for Materials Research (IMO-IMOMEC), Analytical and circular Chemistry (ACC), NMR group, Agoralaan, 3590 Diepenbeek, Belgium

^5^ Sustainable Materials Engineering (SUME), Research Group of Electrochemical and Surface Engineering (SURF), Vrije Universiteit Brussel, Brussels, Belgium

^6^ Centre for Membrane Separations, Adsorption, Catalysis, and Spectroscopy (cMACS), KU Leuven, Leuven, Belgium

† These authors contributed equally to this work and share first authorship

[*** carmela.aprile@unamur.be**](about:blank)

[*** sophie.hermans@uclouvain.be**](about:blank)

[*** wouter.marchal@uhasselt.be**](about:blank)

**Supporting information**

Table S1. Compositional Analysis of zirconia hybrid materials (ICP-AES analysis).

| ***Sample*** | ***Sample Mass(mg)*** | ***Mol of P*** | ***Mol of Zr*** | ***Mol (P/Zr)*** | ***Mol(P/Zr) expected*** |
| --- | --- | --- | --- | --- | --- |
| **Zr-PhDPA-oPA** | 5.2 | 2.998 × E^-5^ ±0.013 | 1.775×E^-5^ ±0.01 | 1.69±0.01 | 1.7 |
| **Zr-PhDPA** | 17.2 | 3.71× E^-5^±0.02 | 8.04 × E^-5^ ±0.04 | 0.46±0.003 | 0.41 |

ICP-AES measurements were performed in duplicate, using digested sample masses ranging from 5 to 20 mg. After digestion, the sample volumes were adjusted to a final volume of 50 mL.


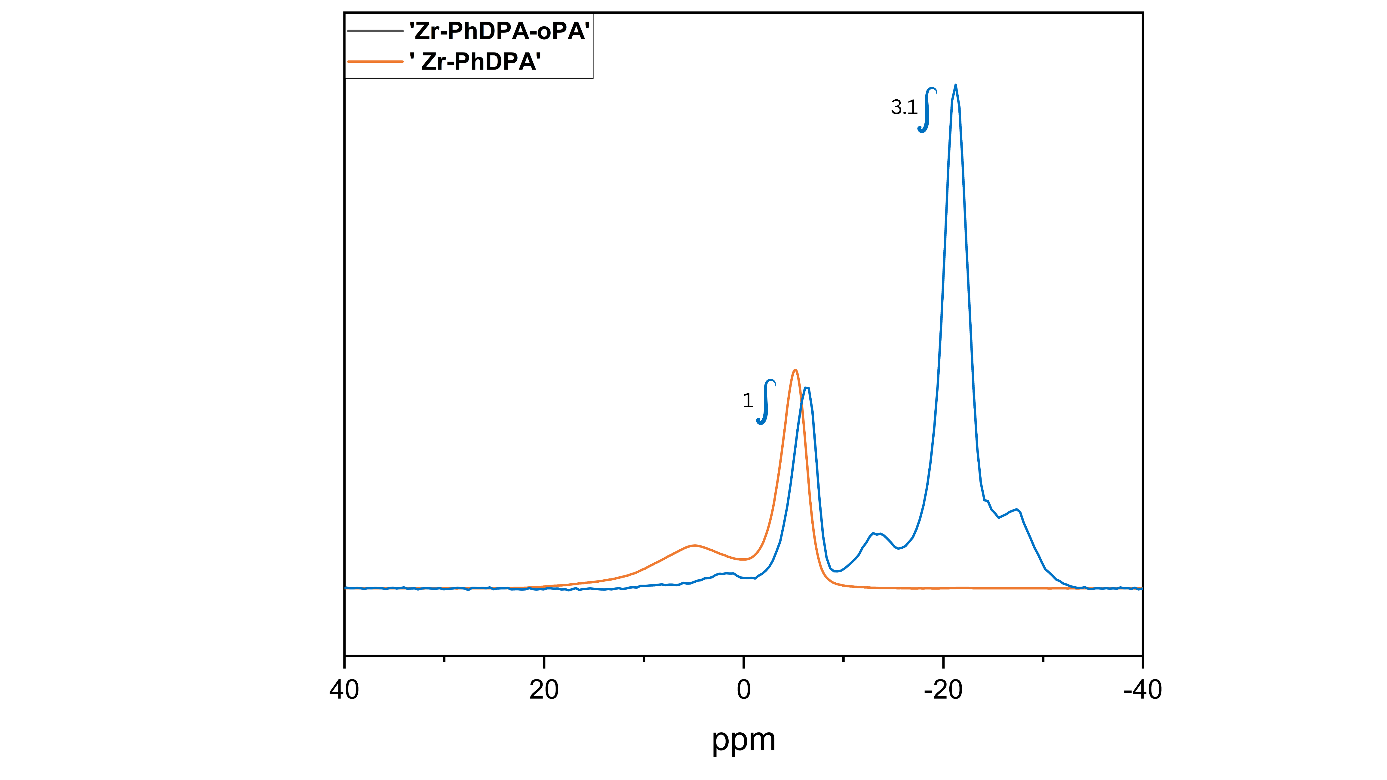


Figure S1. Solid-state ^31^P-MAS NMR spectra of zirconia hybrid materials.





Figure S2. Nitrogen physisorption analysis of zirconia hybrid materials.


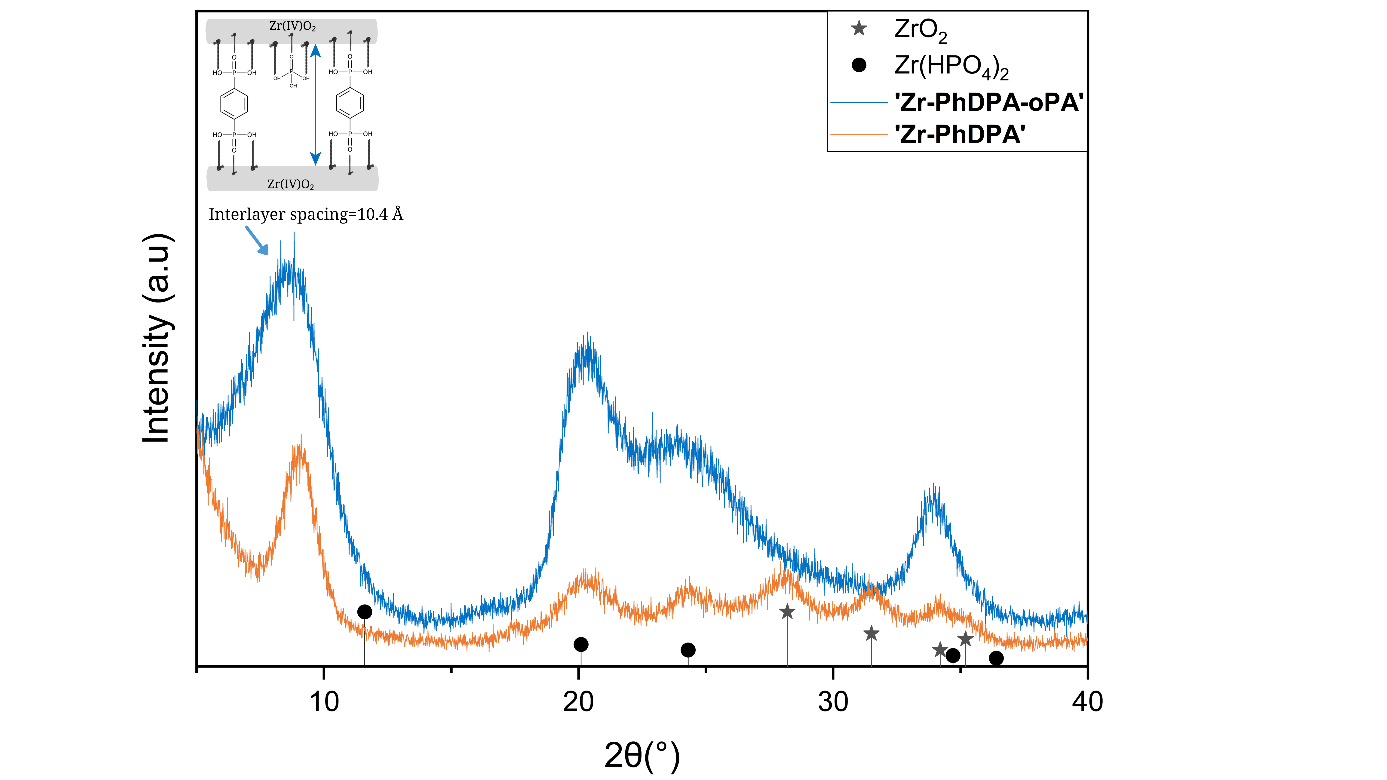


Figure S3. XRD diffractograms of zirconia hybrid materials.


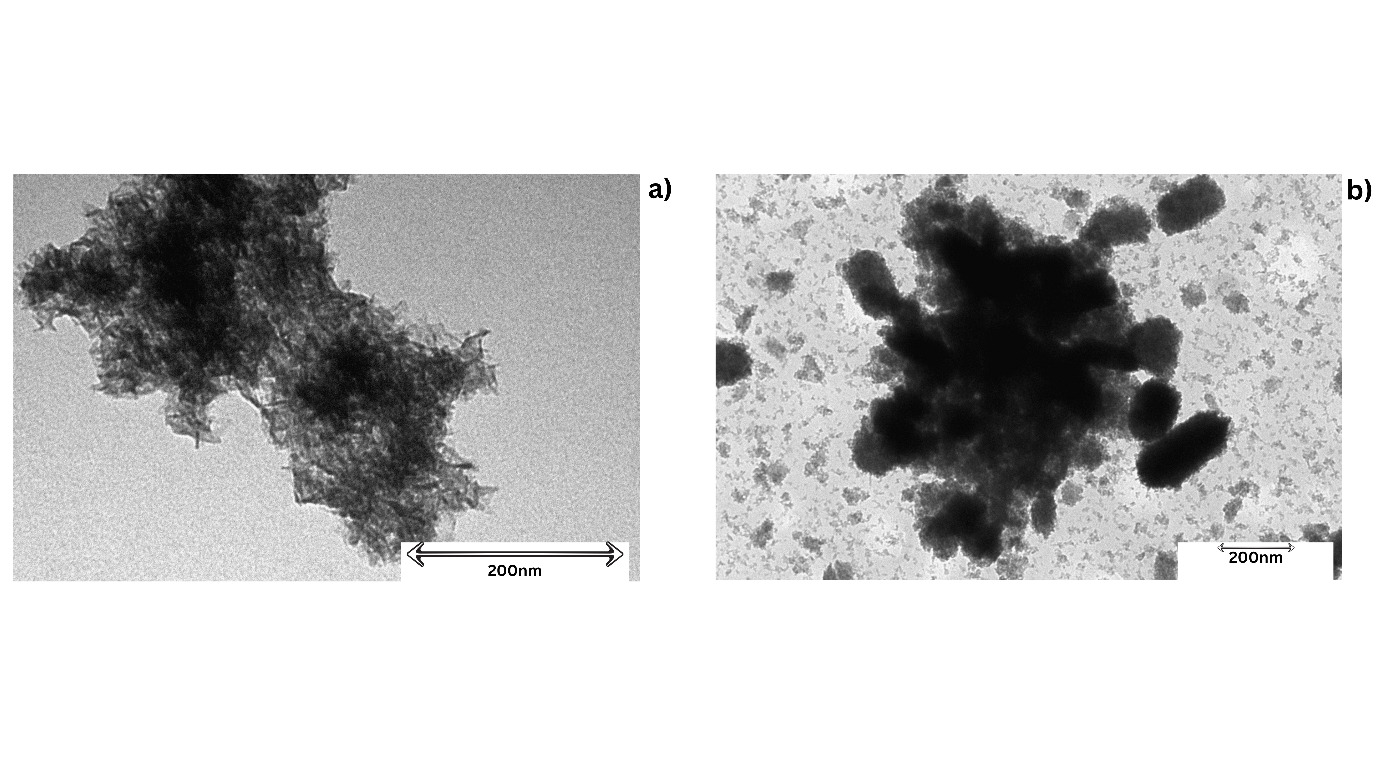


Figure S4**.**TEM image of catalyst ‘Zr-PhDPA-oPA’ (a) and TEM image of catalyst ‘Zr-PhDPA’ (b).


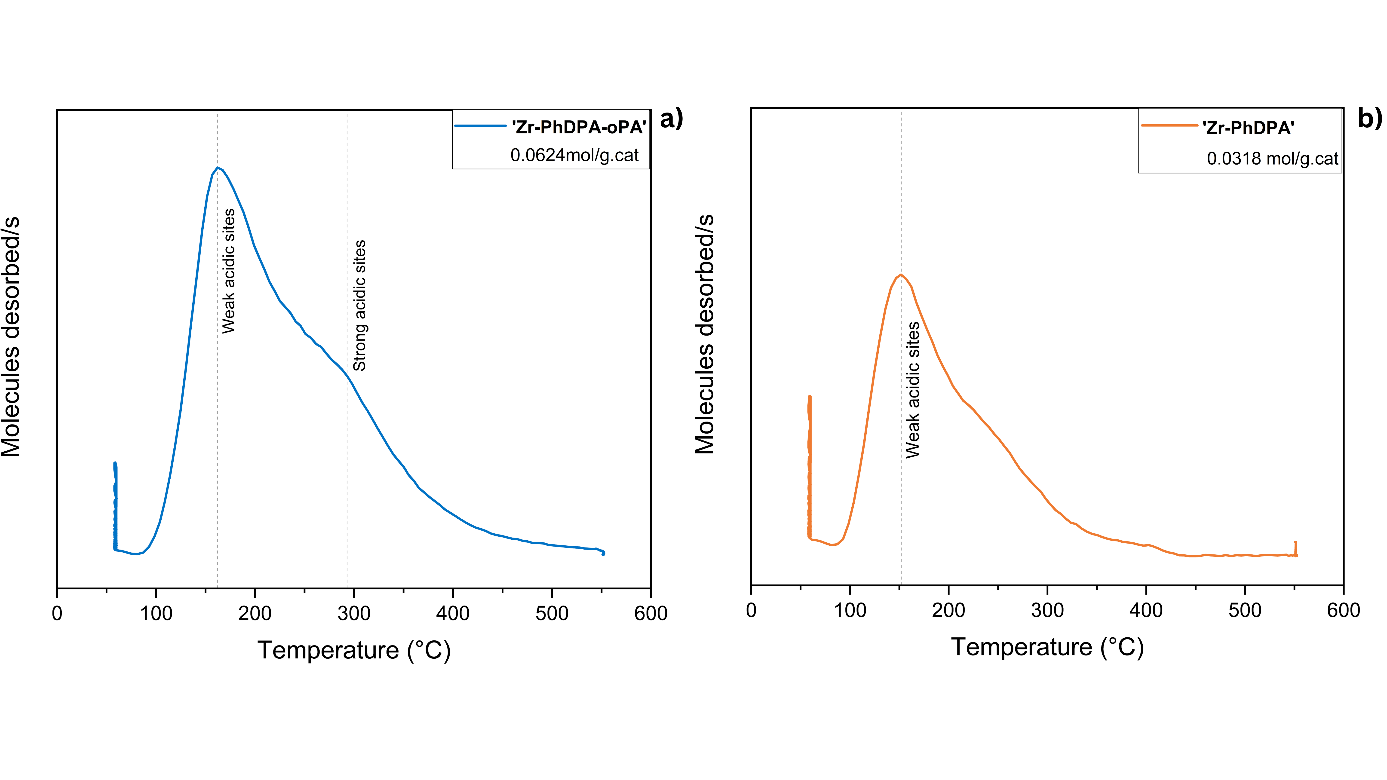


Figure S5. Ammonia TPD acidity profiles of catalyst ‘Zr-PhDPA-oPA’ (a) and Catalyst ‘Zr-PhDPA’ (b).


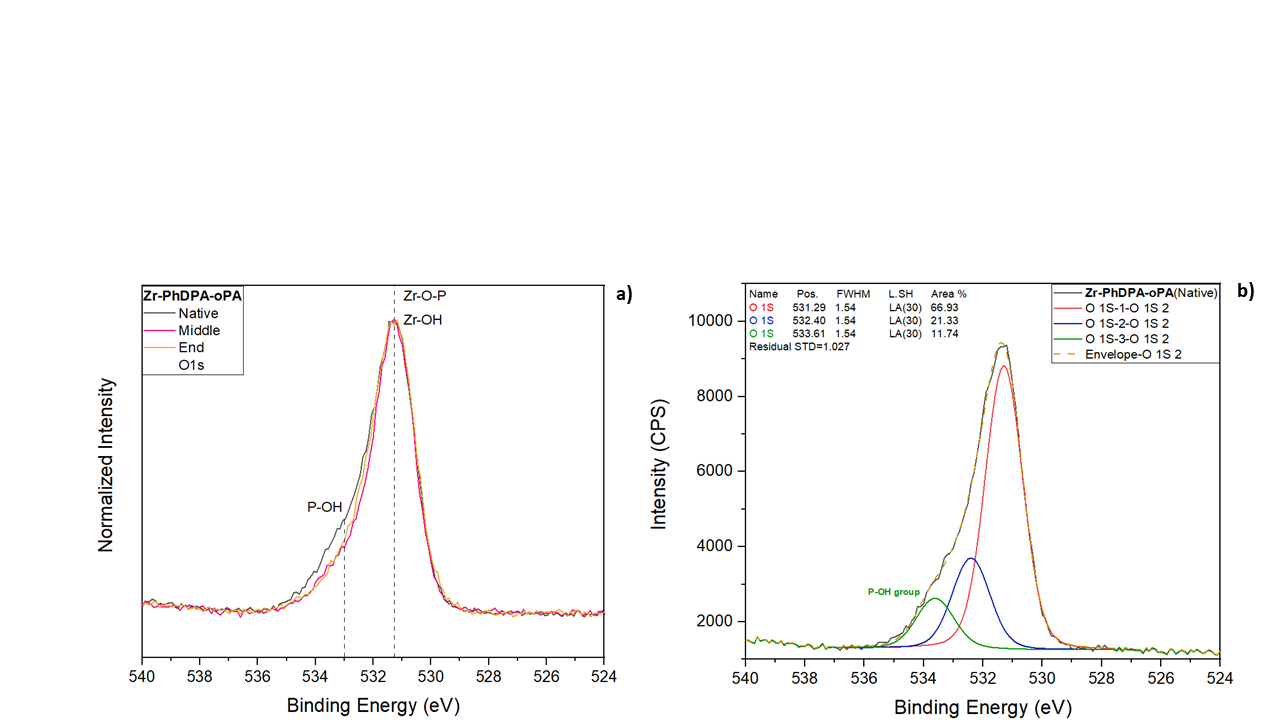


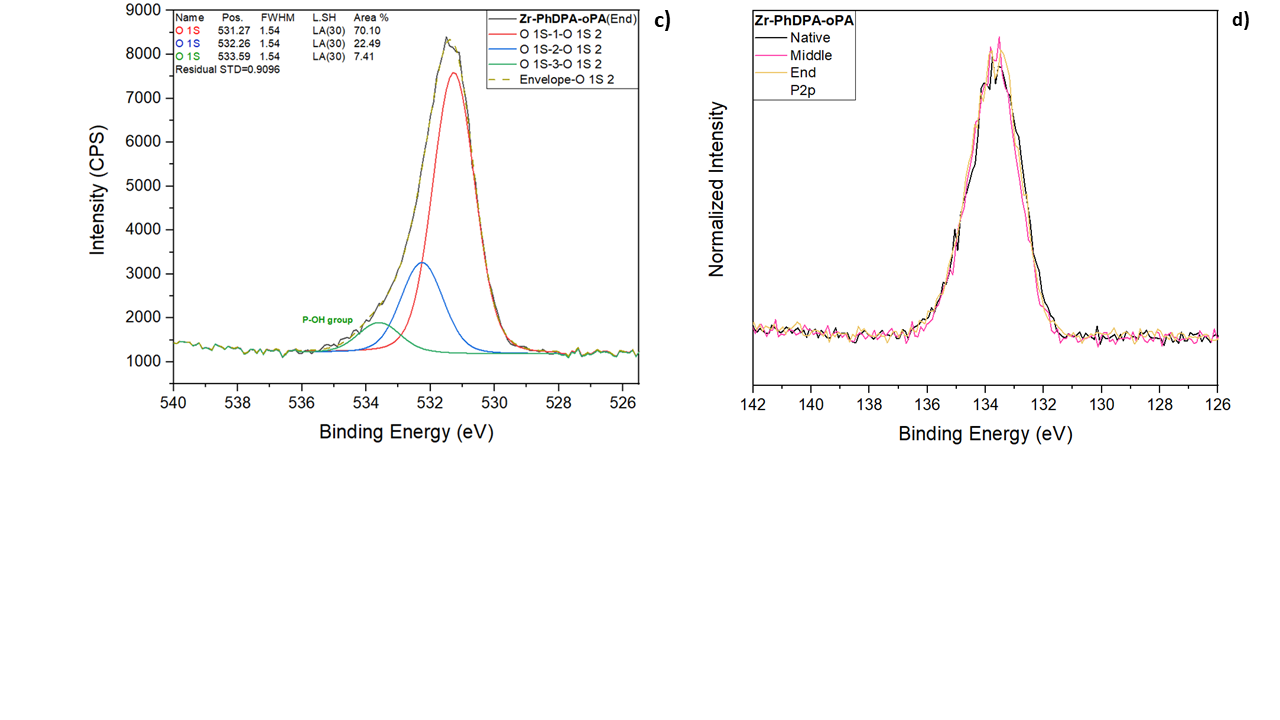


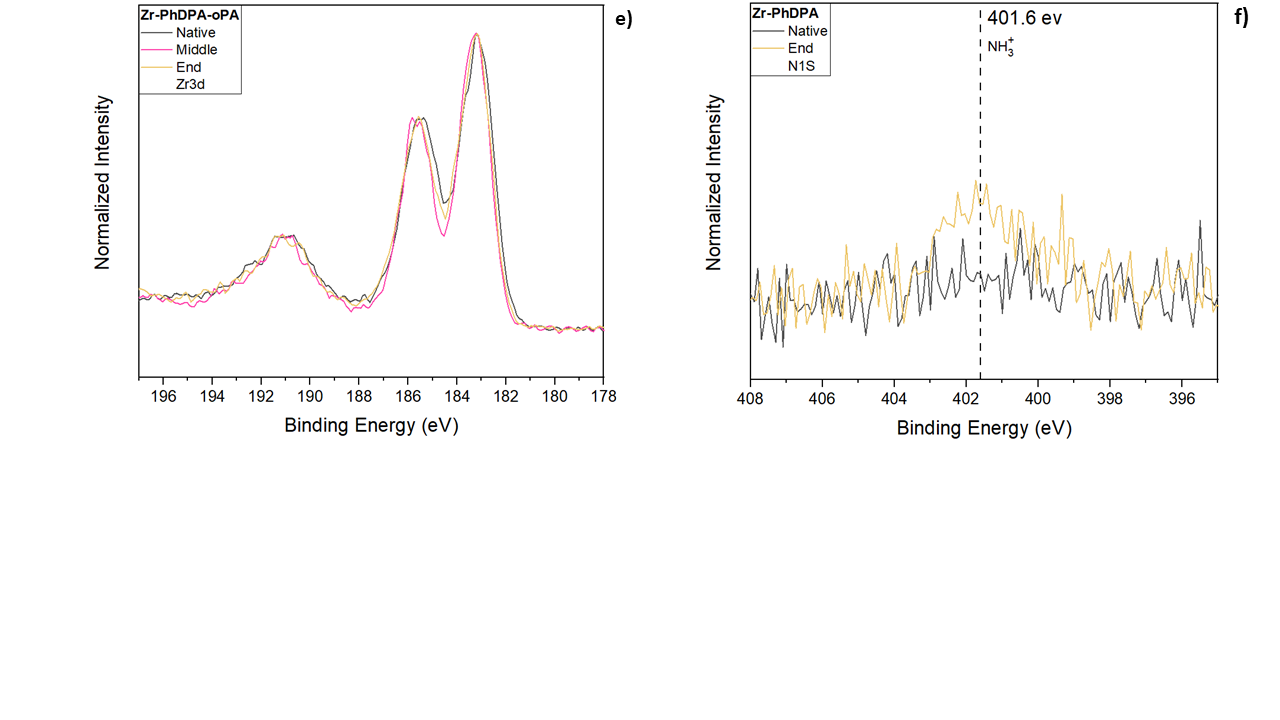


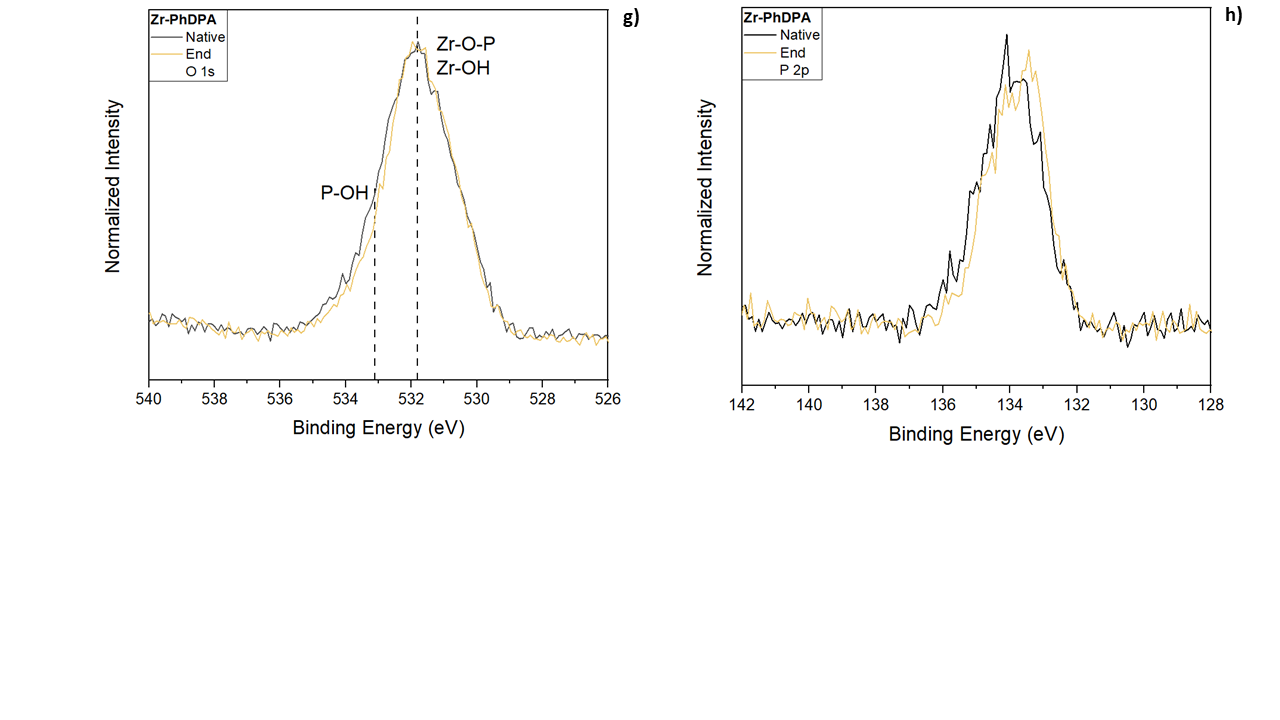

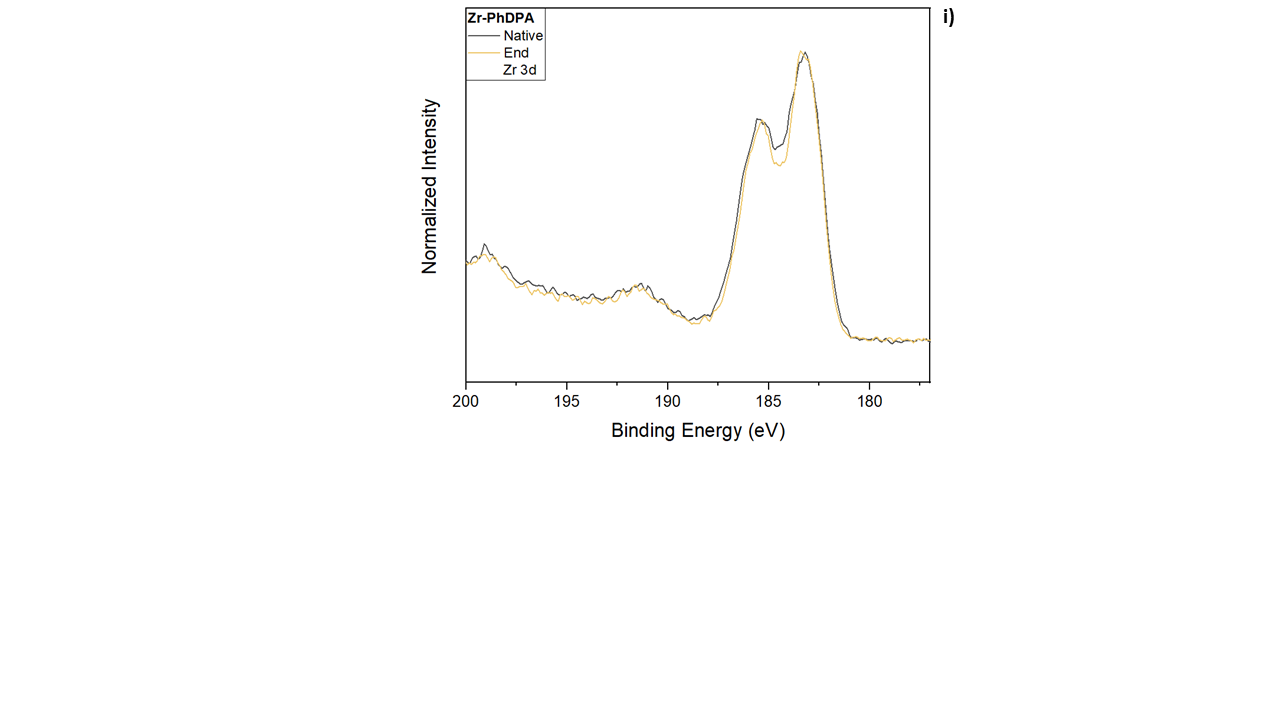


Figure S6. X-ray Photoelectron Spectroscopy (XPS) analysis of catalysts at various titration stages. (a–e) XPS spectra of catalyst ‘Zr-PhDPA-oPA’ at three titration stages: (a) O 1s, (b) fitted O 1s spectrum of the native sample, (c) fitted O 1s spectrum of end sample,(d) P 2p, and (e) Zr 3d. (f–i) XPS spectra of catalyst ‘Zr-PhDPA’ at two titration stages: (f) N 1s, (g) O 1s, (h) P 2p, and (i) Zr 3d.

Table S2. Calculated surface atomic percentages % in catalyst ‘Zr-PhDPA-oPA’ and ‘Zr-PhDPA’ during the titration (XPS analysis).

| ***Sample*** | ***C1s*** |  | ***N 1s*** | ***O 1s*** | ***P 2p*** | ***Zr 3p*** | ***P/Zr*** | ***N/P*** |
| --- | --- | --- | --- | --- | --- | --- | --- | --- |
| ***Zr-PhDPA-oPA*** |  |  |  |  |  |  |  |  |
| **Native** | 19.04 ±0.67 |  | 0.71 ±0.19 | 59.07 ±0.71 | 13.92±0.23 | 7.27 ±0.10 | 1.91 ±0.04 | 0.051 ±0.014 |
| **Middle** | 23.36 ±0.81 |  | 2.25 ±0.33 | 54.84 ±0.36 | 12.75±0.27 | 6.80 ±0.21 | 1.88 ±0.07 | 0.177 ±0.027 |
| **End** | 27.46 ±1.59 |  | 3.55 ±0.12 | 50.29 ±1.25 | 12.36±0.19 | 6.35 ±0.10 | 1.95 ±0.04 | 0.287 ±0.011 |
| ***Zr-PhDPA*** |  |  |  |  |  |  |  |  |
| **Native** | 35.70 ±1.23 |  | 0.38 ±0.30 | 45.12 ±1.16 | 7.28 ±0.46 | 11.52 ±0.40 | 0.63 ±0.05 | 0.052 ±0.041 |
| **End** | 37.11 ±3.14 |  | 1.14 ±0.47 | 43.69 ±1.96 | 6.87 ±0.78 | 11.20 ±0.76 | 0.61 ±0.08 | 0.17± 0.07 |

The atomic percentages in the samples were determined based on average values obtained from multiple measurement spots, confirming the interaction between n-butylamine and the phosphate/phosphonate groups.


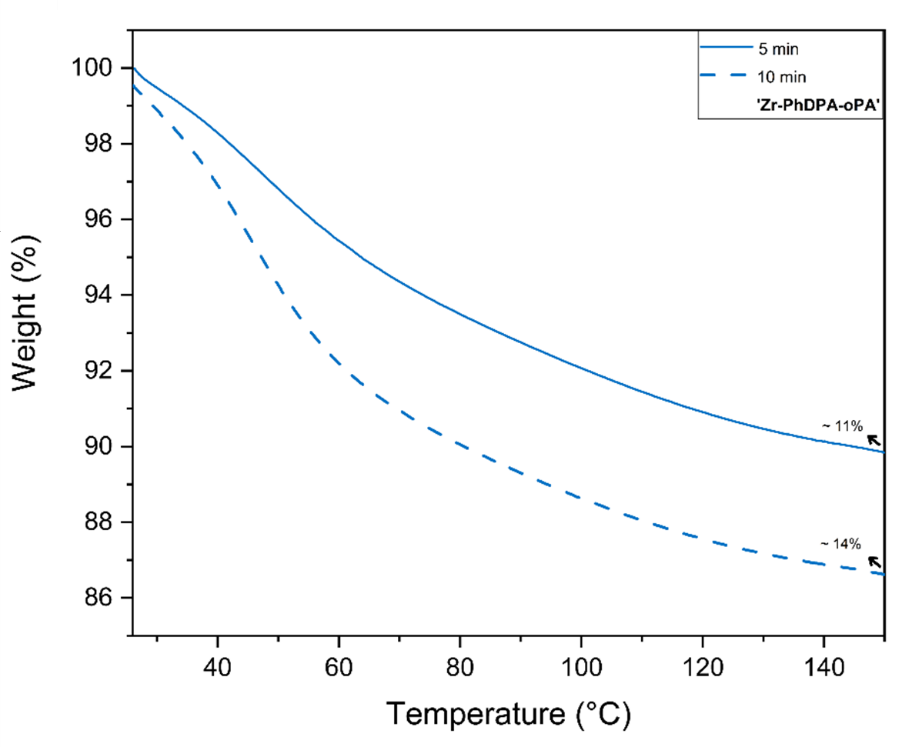


Figure S7. Control thermogravimetric analysis (TGA) experiment in which ‘Zr-PhDPA-oPA’ was exposed to ambient air for 5 and 10 minutes before reheating to 150°C.


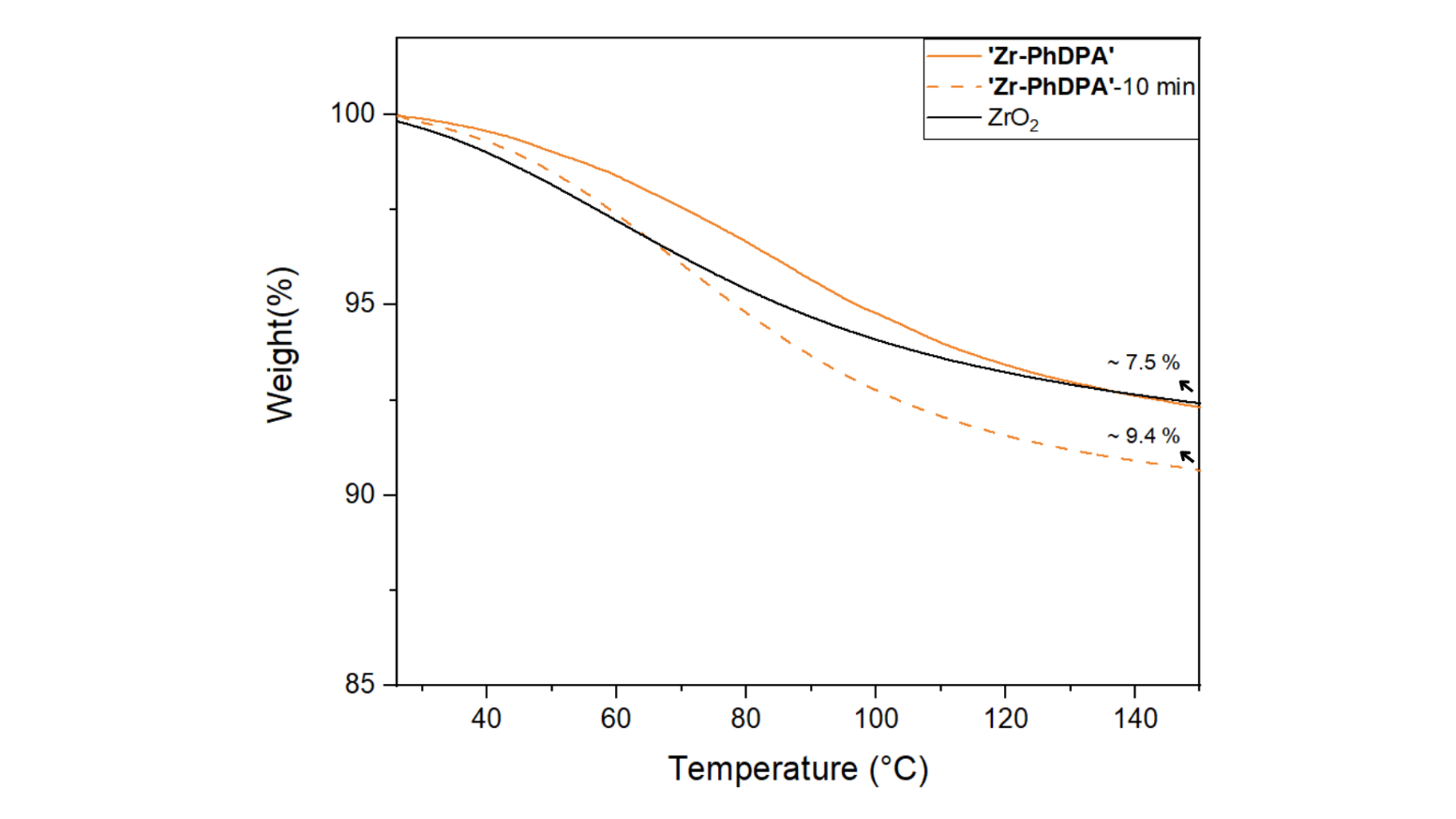


Figure S8. Thermogravimetric analysis (TGA) of the ‘Zr-PhDPA’ sample , the same material after 10 minutes exposure to ambient air before reheating to 150°C (Zr-PhDPA-10 min), and ZrO_2_ as a reference.


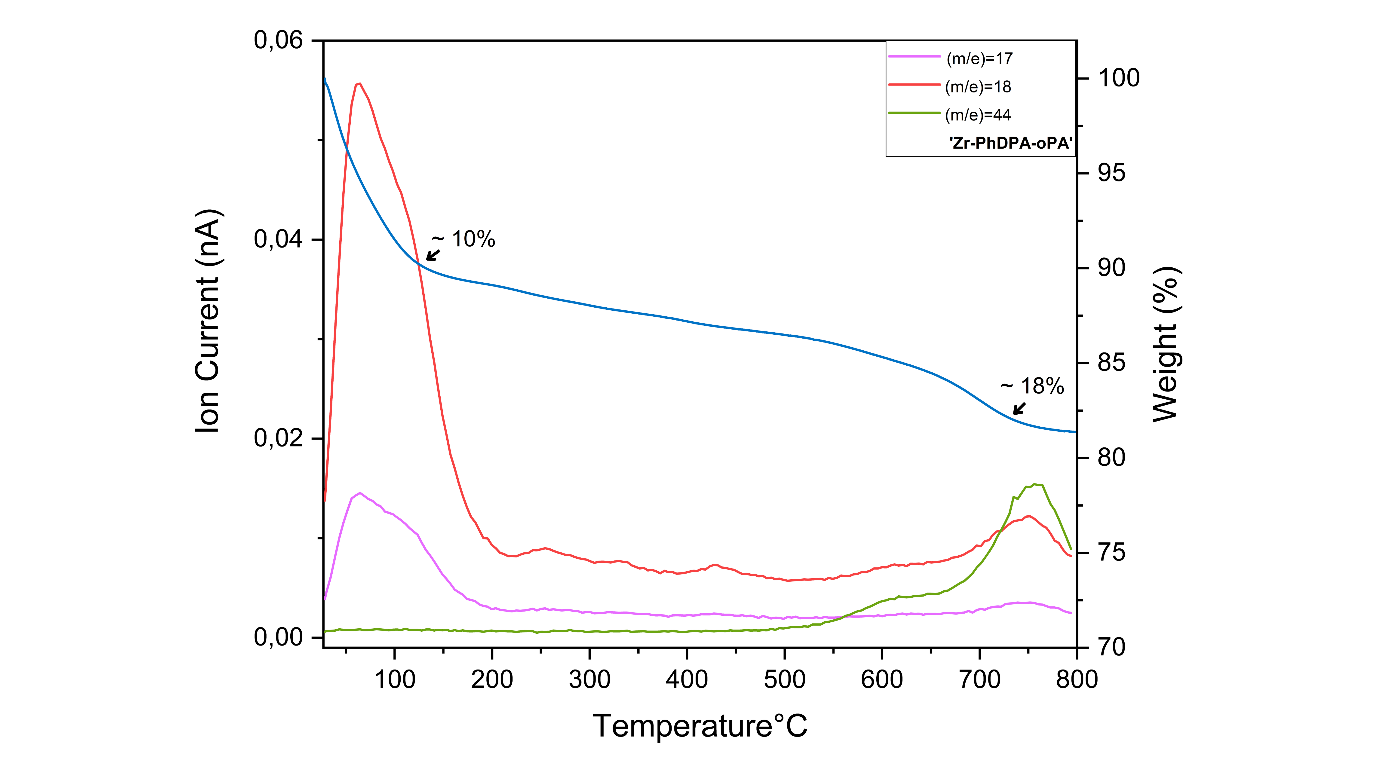


Figure S9. Thermogravimetric analysis coupled with mass spectrometry (TG-MS) of ‘Zr-PhDPA-oPA’.


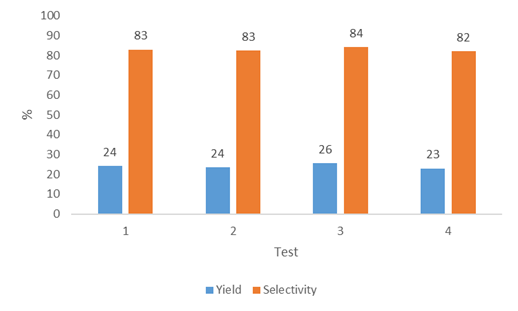


Figure S10. Reproducibility test of Catalyst ‘Zr-PhDPA-oPA’. The catalyst was pre-treated at 60 °C in a vacuum oven overnight before each next test. Conditions of the test: 50 °C, 800 rpm, 0.01 mol glycerol, 0.04 mol acetone, 10 mg catalyst.

**
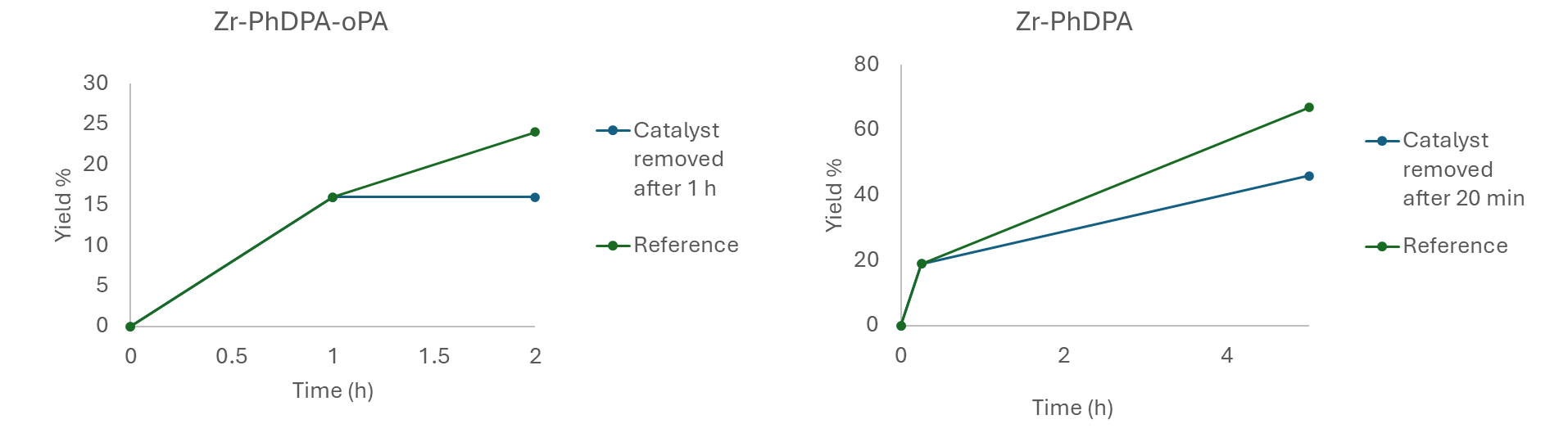
**

Figure S11. Leaching tests of Catalyst ‘Zr-PhDPA-oPA’ and ‘Zr-PhDPA’. Catalysis conditions: both the catalysts were pre-treated at 60 °C in a vacuum oven overnight before testing. Catalyst ‘Zr-PhDPA-oPA’: 50 °C, 800 rpm, 0.01 mol glycerol, 0.04 mol acetone, 10 mg catalyst. Catalyst ‘Zr-PhDPA’: 50 °C, 800 rpm, 0.16 mol glycerol, 0.64 mol acetone, 10 mg catalyst.

**
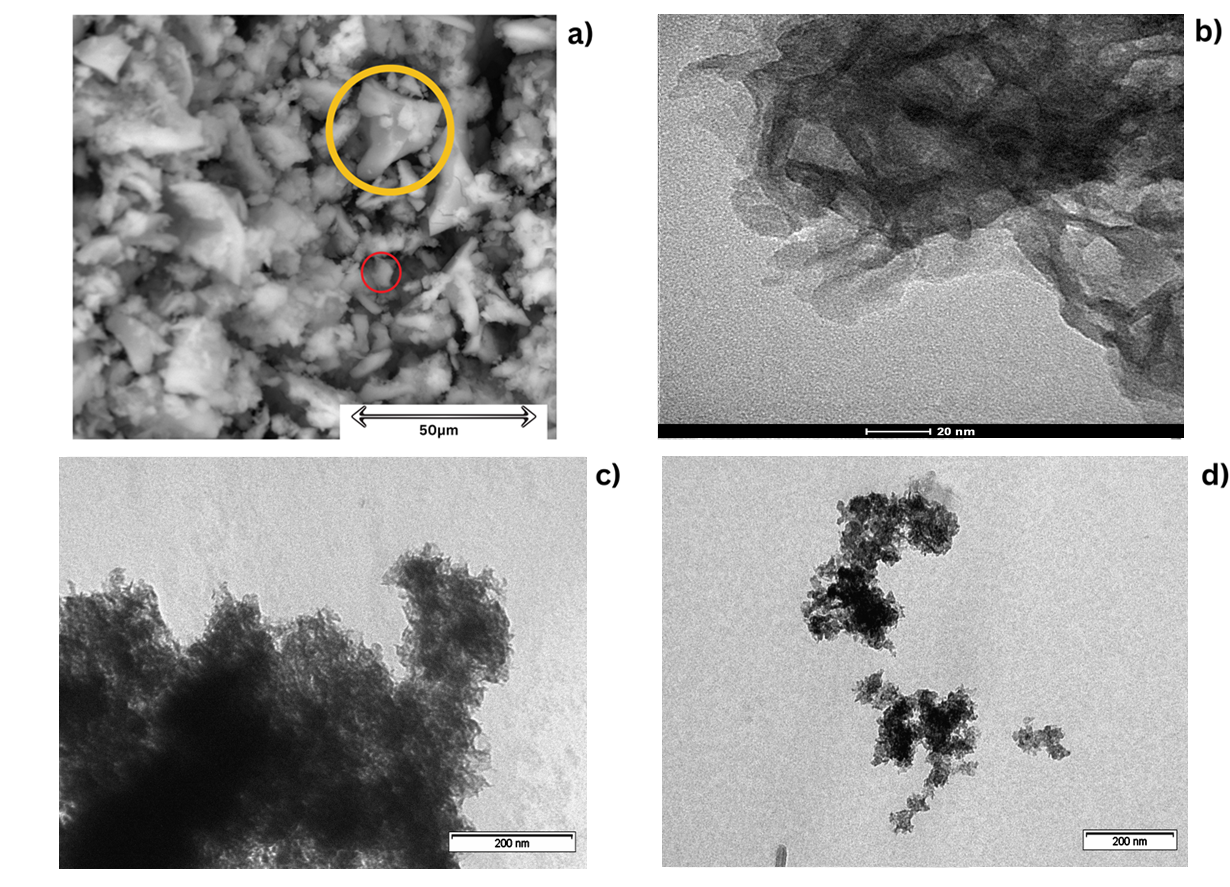
**

Figure S12. SEM pictures of catalyst ‘Zr-PhDPA-oPA’ highlighting the particle size distribiution, with the red circle marking the samller particles and the yellow circle denoting the larger one (a) TEM image of catalyst ‘Zr-PhDPA-oPA’ (high resolution) (b) TEM image of catalyst ‘Zr-PhDPA-oPA’ (c) TEM image of catalyst ‘Zr-PhDPA-oPA’-small (d).


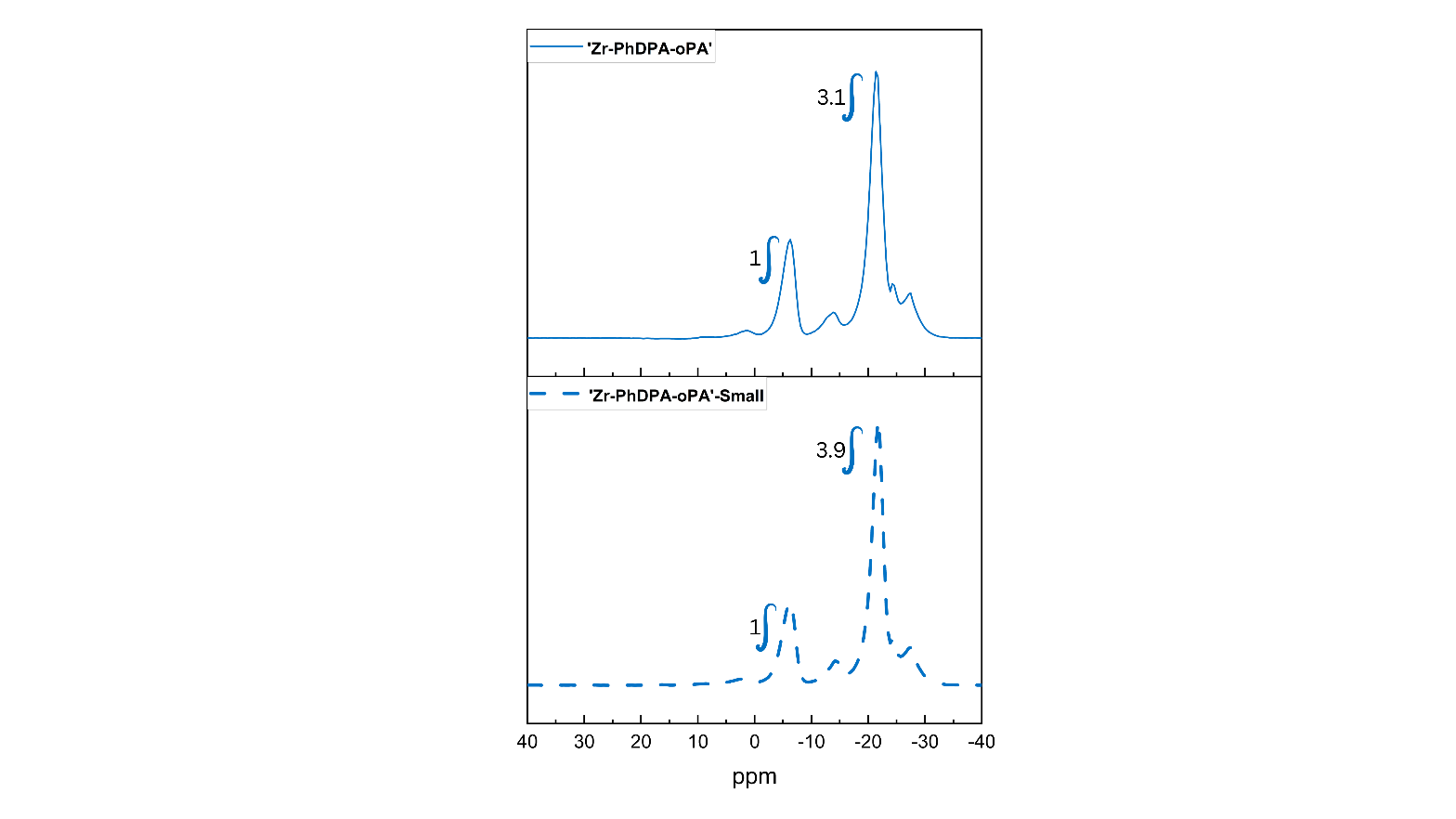


Figure S13. Solid-state ^31^P-MAS NMR spectra of catalyst ‘Zr-PhDPA-oPA’ vs catalyst ‘Zr-PhDPA-oPA’-small.

Table S3. Comparison of Catalyst ‘Zr-PhDPA-oPA’ performances with some materials previously reported in literature. Conditions of the catalytic tests (from entry 1 to 6): 50 °C, 800 rpm, 0.01 mol of glycerol, 0.04 mol of acetone. Conditions of the catalytic tests (from entry 7 and 8): 80 °C, 800 rpm, 0.01 mol of glycerol, 0.01 mol of acetone. Conditions of the catalytic test (entry 9): 50 °C, 0.02 mol of glycerol, 0.2 mol of acetone. Conditions of the catalytic test (entry 10): 40 °C, 0.01 mol of glycerol, 0.04 mol of acetone. ^a^ Vivian A., Soumoy L., Fusaro L., Louette P., Felten A., Fiorilli S., Debecker D.P., Aprile C. Journal of Catalysis, 400 (2021), 83-92. ^b^ Soumoy L., Célis C., Debecker D.P., Armandi M., Fiorilli S., Aprile C. Journal of Catalysis, 411 (2022), 41-53.^c^ Hussein H., Vivian A., Fusaro L., Devillers M., Aprile C. ChemCatChem, 12 (2020), 5966-5976. ^d^ Bivona L.A., Vivian A., Fusaro L., Fiorilli S., Aprile C. Applied Catalysis B: Environmental, 247 (2019), 182-190. ^e^ Li L., Korányi, T. I., Sels B. F., Pescarmona, P. P. Green Chemistry, 14 (2012), 1611-1619. ^f^ Li X., Jiang Y., Zhou R., Hou Z. Applied Clay Science, 174 (2019), 120-126. ^g^ Li X., Jiang Y., Zhou R., Hou Z. Applied Clay Science, 189 (2020), 105555.

| **Entry** | **Catalyst** | **Time  (h)** | **Mass of catalyst (mg)** | **Yield  (%)** | **Productivity/time**  **(h^-1^)** | **Reference** |
| --- | --- | --- | --- | --- | --- | --- |
| 1 | Zr-PhDPA-oPA | 2 | 10 | 27 | 18 | This work |
| 2 | Zr-PhDPA-oPA-small | 2 | 10 | 41 | 27 | This work |
| 3 | Ga-37 | 2 | 10 | 30 | 20 | Vivian et al.^a^ |
| 4 | Hf-Acac-NT-c | 2 | 10 | 26 | 17 | Soumoy et al.^b^ |
| 5 | XS-GaLac-A | 3 | 10 | 25 | 11 | Hussein et al.^c^ |
| 6 | Sn-NTs-B | 2 | 25 | 24 | 6 | Bivona et al.^d^ |
| 7 | Zr-TUD-1 | 6 | 25 | 46 | 4 | Li et al.^e^ |
| 8 | Hf-TUD-1 | 6 | 25 | 52 | 5 | Li et al.^e^ |
| 9 | ZrP-200 | 3 | 100 | 84 | 8 | Li et al.^f^ |
| 10 | ZrPP-20 | 6 | 50 | 76 | 4 | Li et al.^g^ |
|  |  |  |  |  |  |  |
